# Discovery of antimicrobial peptides from incomplete biosynthetic gene clusters to combat multidrug-resistant bacteria

**DOI:** 10.1016/j.xcrm.2026.102982

**Published:** 2026-08-12

**Authors:** Yao Tang, Xin Cheng, Jing Zhou, Yuqi Shi, Jiaying Zhou, Zaiyang Chen, Yan Zhang, Liucun Zhu, Jian-Qun Chen, Jiang Gu, Qiang Wang, Qihan Chen

**Affiliations:** 1 MOE Frontiers Science Center for Precision Oncology, University of Macau, Macau SAR, China; 2 Faculty of Medicine, University of Macau, Macau SAR, China; 3 Department of Thoracic Surgery, Nanjing Drum Tower Hospital, Affiliated Hospital of Medical School, Nanjing University, Nanjing, Jiangsu 210093, China; 4 National Engineering Research Center of Immunological Products, Department of Microbiology and Biochemical Pharmacy, College of Pharmacy, Army Medical University, Chongqing 400038, China; 5 The State Key Laboratory of Pharmaceutical Biotechnology, School of Life Sciences, Nanjing University, Nanjing, Jiangsu 210023, China; 6 Department of Clinical Laboratory, Nanjing Drum Tower Hospital, Affiliated Hospital of Medical School, Nanjing University, Nanjing, Jiangsu 210093, China; 7 School of Life Sciences, Shanghai University, Shanghai 200444, China

**Keywords:** antimicrobial peptide, antibiotic discovery, polymyxin, nonribosomal peptides, multidrug-resistant bacteria

## Abstract

The escalating crisis of multidrug-resistant bacteria necessitates innovative antibiotic discovery platforms. Conventional antimicrobial peptide (AMP) mining often relies on complete biosynthetic gene clusters (BGCs), leaving fragmented genomic resources underexplored. Here, we present an evolution-inspired approach to reconstruct and predict AMPs from partial BGCs. Applying this strategy to 954 *Paenibacillus* genomes identifies five polymyxin-like peptides, NP001–NP005, with broad *in vitro* activity. Crucially, in murine models of polymyxin-resistant infection, NP001 reduced bacterial burdens by up to 1,000-fold in a thigh infection model and improved survival (50% vs. 0%) in a lethal peritonitis model. Structural simulations and biophysical assays revealed that NP001 maintains high affinity for bacterial membranes and effectively binds to *MCR*-*1*-modified lipid A, a key colistin-resistance mechanism. Moreover, Leu at position 10 of NP001 plays a key role in antibacterial activity against *MCR*-1-resistant bacteria. Our work establishes a generalizable framework for AMP discovery and introduces a promising therapeutic candidate, NP001, which effectively counteracts polymyxin-resistant pathogens.

## Highlights

• An evolution-guided algorithm broadly identifies NRPs from incomplete BGCs
• *Paenibacillus* genomes yield five polymyxin-class antimicrobial peptides (NP001–005)
• NP001 is active against Gram-negative bacteria *(MCR-1)* in mice
• NP001 binds to bacterial membranes and lyses cells; residue 10 plays a key role

## Graphical abstract

**Figure:**
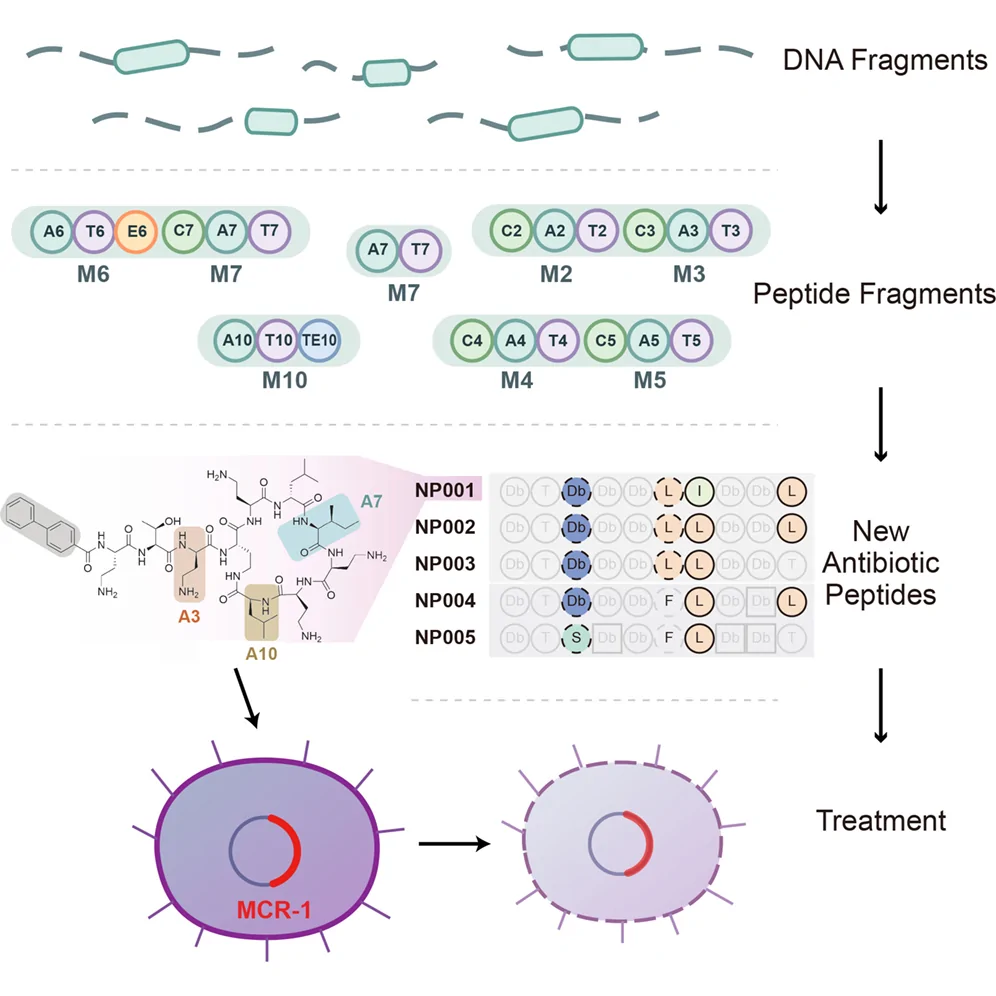

## Teaser

Tang et al. develop an evolution-guided strategy to mine nonribosomal peptide synthetase (NRPS) peptides from genomic fragments, yielding five new antibacterial polymyxin analogues in *Paenibacillus*. Among these, NP001 effectively inhibits Gram-negative bacterial strains containing *MCR-1 in vitro* and *in vivo*, indicating that it is a promising drug candidate.

## Introduction

The global threat of multidrug-resistant Gram-negative bacteria (MDR-GNB) has escalated into a critical public health crisis, with pathogens such as carbapenem-resistant *Enterobacteriaceae*, *Acinetobacter baumannii*, and *Pseudomonas aeruginosa* listed as priority organisms by the World Health Organization.^1^^,^^2^^,^^3^ The depletion of effective therapeutics has positioned natural antimicrobial peptides (AMPs) derived from *Paenibacillus* spp. polymyxins, including colistin, as last-line defenses. These agents target lipid A within lipopolysaccharides (LPSs), disrupting the integrity of the outer membrane of Gram-negative bacteria. However, the emergence and global dissemination of plasmid-encoded resistance genes (e.g., *MCR-1*) have severely compromised polymyxin efficacy, threatening this final therapeutic option.^4^^,^^5^ Consequently, the development of new polymyxin analogues capable of overcoming existing resistance represents an urgent and unmet medical need.

Traditional routes such as microbial metabolite purification or structural optimization face considerable limitations in discovering polymyxin analogues. Natural product isolation is hampered by low throughput,^3^ while rational chemical modification struggles to decouple therapeutic efficacy from nephrotoxicity.^6^ In response, genomics-guided discovery has emerged as a powerful alternative. Recent studies have successfully identified novel AMPs through strategies including small open reading frame mining,^7^ broad-spectrum genomic screening,^8^ high-throughput peptide synthesis,^9^ and database-driven prioritization.^10^ These advances underscore the potential of computational biology to revitalize antibiotic discovery.

Despite this progress, a fundamental dependency on complete and well-assembled biosynthetic gene clusters (BGCs) constrains most current approaches. Nonribosomal peptides (NRPs) like polymyxins are synthesized by multi-modular BGCs, and tools such as antiSMASH require largely contiguous genomic loci for accurate prediction.^11^ In practice, however, the pervasive fragmentation of bacterial genomes leaves the majority of BGCs incomplete or unannotated due to sequencing and assembly limitations.^12^ This largely untapped reservoir of partial biosynthetic information represents a major missed opportunity. Therefore, we aim to establish a broadly applicable strategy for the sequence reconstruction of nonribosomal peptide synthetase (NRPS)-derived AMPs, to enable the mining of previously unidentified candidate peptides from incomplete BGCs. Given that MCR-mediated polymyxin resistance has become a pressing issue in the treatment of multidrug-resistant Gram-negative bacteria, this study selects polymyxin-class peptides as the primary targets for mining and validation. Consequently, polymyxin-class BGCs provide a model with clear clinical significance in this study to evaluate whether incomplete NRPS BGCs can be transformed into synthesizable and verifiable candidate AMPs.

## Results

### A phylogenetics-based strategy for mining NRPs from partial BGCs

**Figure 1:**
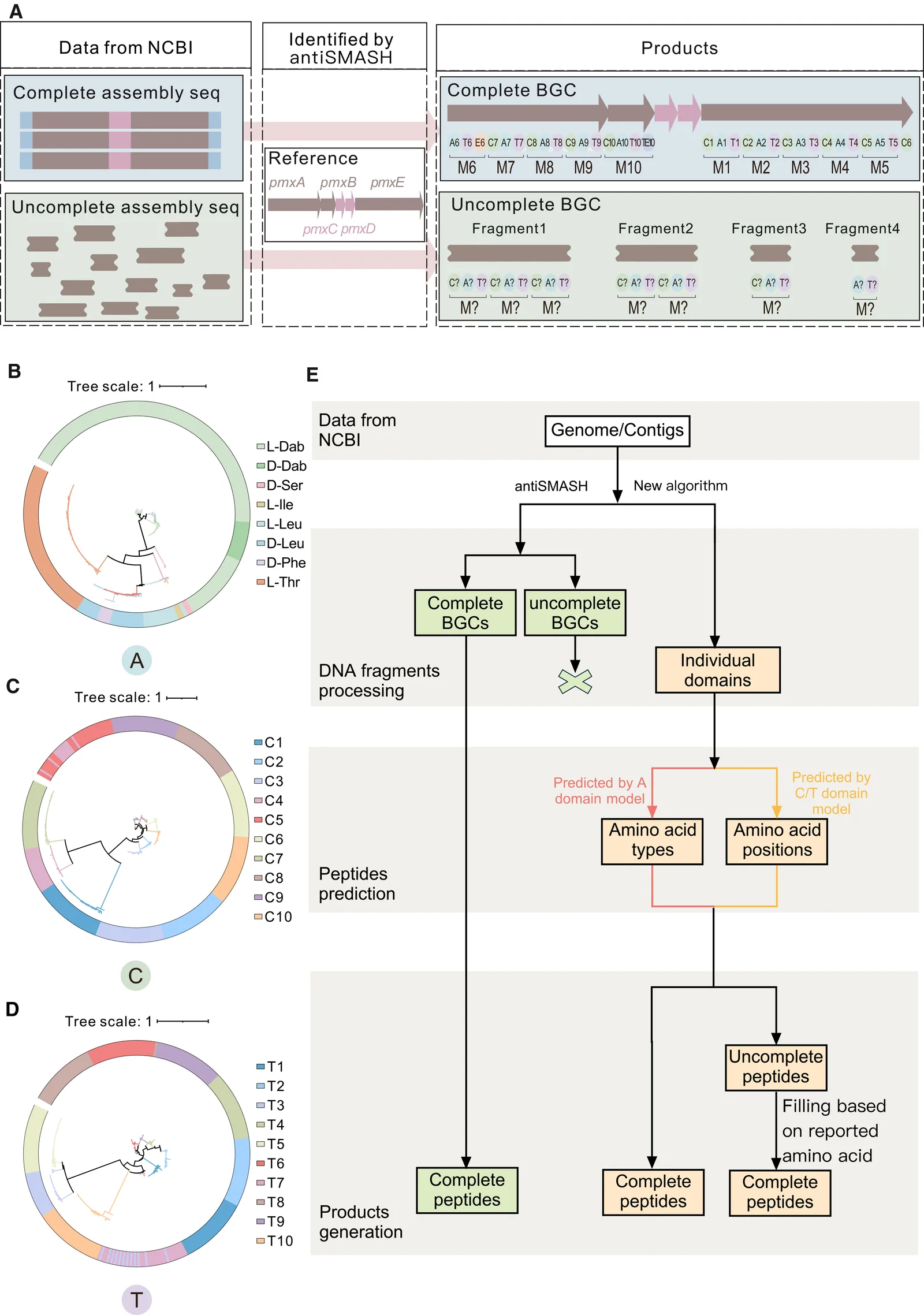
An evolution-guided NRPS reconstruction workflow (A) Schematic representation of existing workflows for peptide identification. Taking the identification of polymyxin using the antiSMASH tool as an example, when a complete BGC is identified, the A domain can be used to determine the substrate type, and the sequential order of modules allows for the prediction of amino acid combinations. However, when an incomplete BGC is identified, the precise positions of the modules cannot be determined, preventing accurate prediction of the resulting peptide product. (B) Phylogenetic tree of A domains. A phylogenetic tree was constructed based on the amino acid sequences of A domains, with annotations indicating the amino acid substrates recognized by each domain. The tree demonstrates that different amino acid types cluster within distinct branches. (C and D) Phylogenetic trees of C and T domains. Phylogenetic trees were constructed based on the amino acid sequences of C and T domains, with annotations indicating their module positions within BGCs. The trees show that C and T domains at different positions cluster into distinct branches. (E) Comparison between the antiSMASH approach and evolution-guided NRPS reconstruction workflow developed in this study.

NRPs are synthesized by modular BGCs, in which each minimal module—comprising adenylation (A), thiolation (T), and condensation (C) domains—incorporates a specific amino acid into the growing peptide chain. Conventional NRP discovery pipelines, such as those using antiSMASH, require the identification of complete BGCs to predict the final peptide product by comparing them with reference clusters (e.g., polymyxin BGCs in the MIBIG database) and by analyzing A domain specificities (Figure 1A). However, this approach fails when genomic data are fragmented, as incomplete BGCs preclude the accurate determination of amino acid order and cyclization sites, rendering many potential NRP candidates undiscoverable.

To overcome this limitation, we developed a phylogenetics-based strategy that operates on individual biosynthetic domains, rather than complete BGCs. We began by analyzing 954 *Paenibacillus* genomes, from which antiSMASH identified 35 polymyxin BGCs with well-characterized peptide products as a high-confidence reference dataset. We then extracted the sequences of A, C, and T domains from these BGCs to establish two essential relationships: (1) which amino acid a given A domain recognizes, and (2) where that amino acid is positioned in the final peptide.

Phylogenetic analysis of the A domain sequences confirmed that their evolutionary clustering strongly correlates with substrate specificity, as expected from the deterministic nature of A domain structure (Figure 1B; Figure S1A). The key innovation of our method lies in deciphering positional information from the C and T domains. We constructed phylogenetic trees for both C and T domain sequences and found that each formed ten distinct clades. Notably, these clades corresponded precisely to the ten modular positions in the decapeptide polymyxin scaffold, rather than to the identity of the incorporated amino acids (Figures 1C and 1D; Figures S1B and S1C). This demonstrates that C and T domains carry a phylogenetic signature of their positional role within the NRPS assembly line.

This discovery enables the prediction of functional peptides from fragmented genomic data. Provided a genomic fragment contains at least one A domain alongside its adjacent C or T domains, we can now determine both the amino acid identity and its absolute position in the final product, enabling the reconstruction of partial or complete NRPs (Figure 1E).

### Identification of unreported polymyxin analogues from genomic fragments

Having established that our phylogenetic method can decode amino acid identity and position from fragmented BGCs, we applied it to the 954 *Paenibacillus* genomes.

**Figure 2:**
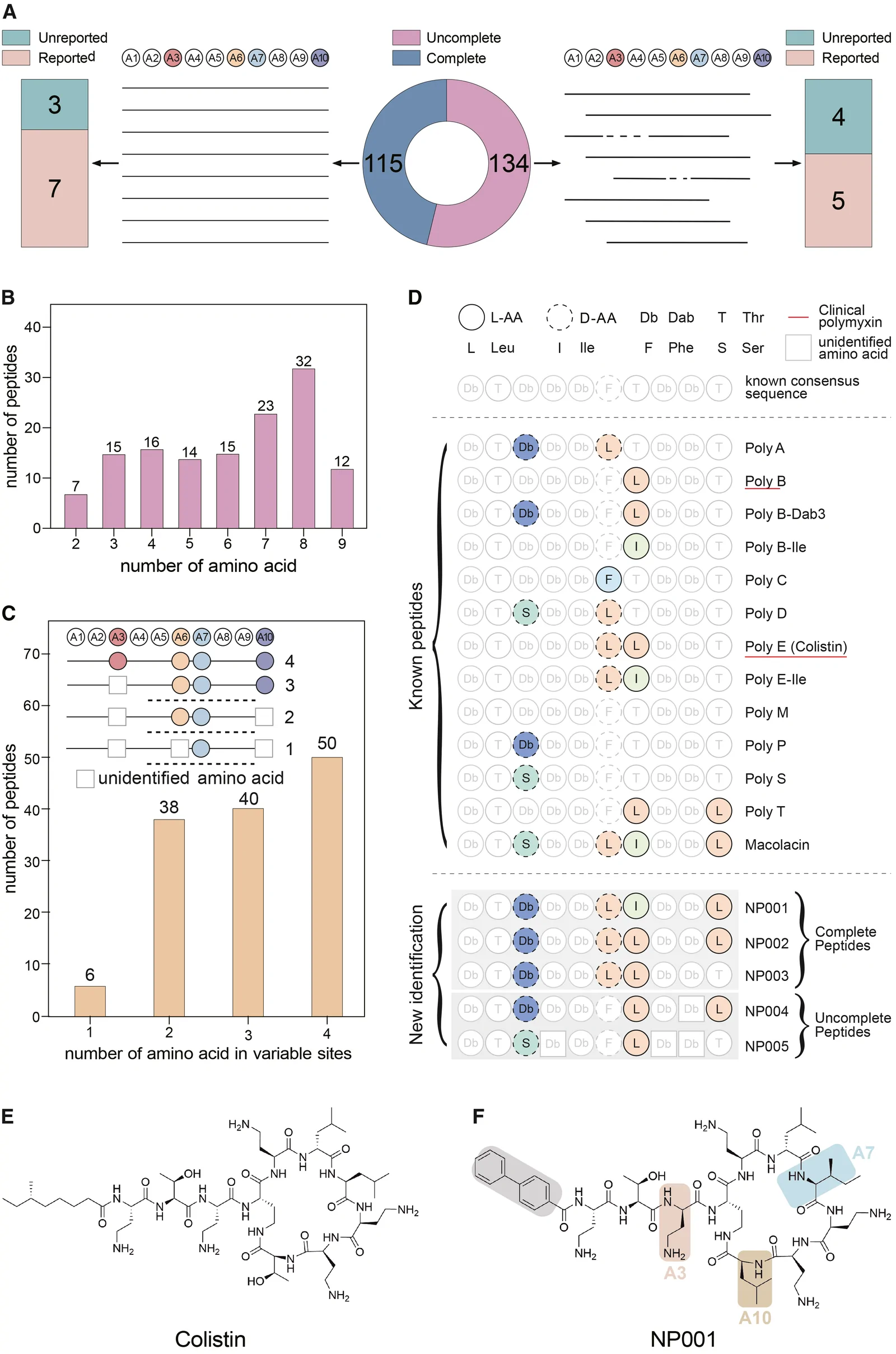
Identification of antimicrobial peptides (A) Distribution of antimicrobial peptides identified in this study. From the 954 *Paenibacillus* genomes, a total of 115 complete peptide molecules (left) and 134 uncomplete peptides (right) were identified. In the sequences, variant amino acid sites are highlighted in colors, and conserved amino acid sites are shown in black. (B) Amino acid count in incomplete peptides. The *x* axis represents the length of amino acids in incomplete peptides, while the *y* axis represents the number of identified antimicrobial peptides. (C) Distribution of missing residues occurring at variant positions (A3, A6, A7, or A10) in incomplete peptides. (D) Sequences of newly identified antimicrobial peptides. Top: sequences of known polymyxins and their analogs; bottom: identified antimicrobial peptide sequences previously unreported. After eliminating sequences that shared identical variant positions with those in complete and incomplete peptides, 5 antimicrobial peptide sequences were identified. (E and F) Structures of polymyxin E (colistin) and NP001. Distinct amino acid positions in both antimicrobial peptides are highlighted with colored backgrounds.

From complete BGCs, we identified 10 distinct peptides, three of which were unreported polymyxin analogues (Figure 2A). These unreported peptides exhibited sequence variations specifically within the known variable regions of polymyxins (Table S1). Critically, our method also enabled the mining of incomplete BGCs. We successfully predicted polypeptide sequences from fragments containing as few as seven amino acids. Over 50% of these fragments covered at least three of the four key variable positions (A3, A6, A7, and A10) that define polymyxin diversity (Figures 2B and 2C; Table S1). By complementing the missing conserved regions and removing duplicates, we reconstructed nine distinct peptides from fragmented data alone. Four of these were unreported discoveries (Figures 2A and 2D).

After integrating the results from complete (NP001–003) and fragmented peptides analyses (NP001 and 003–005) and removing duplicated candidates, five previously unreported peptides (NP001–NP005) were identified (Figure 2D; Table S2). As a representative example, a genomic fragment from *Paenibacillus barcinonensis* KACC11450 (NCBI Refseq: GCF_013347305.1) was decoded to predict a polymyxin-like peptide with a distinct residue profile (D-Dab, D-Leu, Ile, and Leu) at variable positions A3, A6, A7, and A10, differing from colistin at three sites (Figures 2E and 2F). N-terminal acyl substituents are important structural elements of lipopeptide antibiotics, as they can influence hydrophobicity, membrane binding capacity, membrane insertion efficiency, and *in vivo* antibacterial activity. Wang et al. previously performed N-terminal acyl optimization on macolacin, and their results showed that different N-terminal acyl substitutions could significantly affect its antibacterial activity. Among these substitutions, the biphenyl acyl variant exhibited potent *in vitro* activity against polymyxin-resistant bacteria and retained *in vivo* efficacy in a mouse infection model.^4^ Based on this precedent, we synthesized NP001–NP005 with a uniform N-terminal biphenyl acyl chain to evaluate the antibacterial potential of the reconstructed polymyxin-class peptide cores (Figures 2E and 2F; Figure S3). Notably, the use of a biphenyl acyl chain as the N-terminal substituent is not a universal modification that generally enhances the activity of all polymyxin-class peptides, and its effects may vary depending on the peptide sequence. Given that these peptides are products of natural evolutionary processes, we hypothesized that they may possess inherent activity against contemporary polymyxin-resistant pathogens.

### Unreported peptides overcame polymyxin resistance *in vitro*

**Figure 3:**
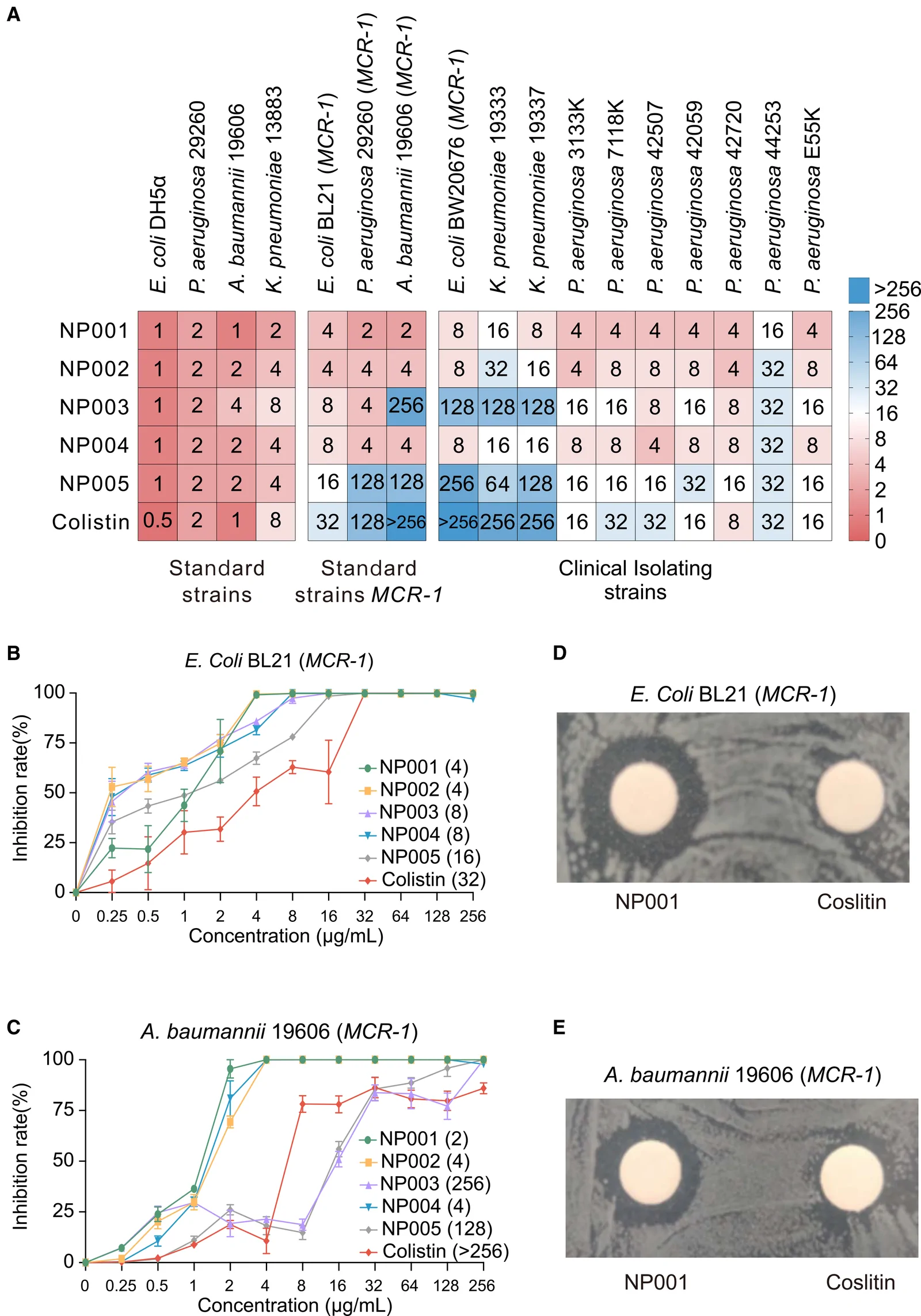
*In vitro* antibacterial activity of antimicrobial peptides (A) Minimum MIC values of the six tested antimicrobial peptides. (B and C) Growth inhibition of *E. coli* BL21 (*MCR-1*) (B) and *A. baumannii* 19606 (*MCR-1*) (C) at different antimicrobial peptide concentrations. All MIC assays were performed with three biological replicates. Error bars represent the SEM. (D and E) Disk diffusion antimicrobial susceptibility test of *E. coli* BL21 (*MCR-1*) (D) and *A. baumannii* 19606 (*MCR-1*) (E).

With the five unreported peptides (NP001–NP005) synthesized and characterized (Figure S3), we first evaluated their antibacterial activity against a panel of standard Gram-negative pathogens, including *Klebsiella pneumoniae*, *Acinetobacter baumannii*, *Pseudomonas aeruginosa*, and *Escherichia coli*. All five peptides exhibited potent activity, with minimum inhibitory concentration (MIC) values comparable to or lower than those of colistin (Figure 3A; Figure S4).

We next challenged the peptides against polymyxin-resistant strains engineered to express *MCR-1* (Table S4). While colistin efficacy was severely compromised (MICs = 32 to >256 μg/mL), three peptides (NP001, NP002, and NP004) demonstrated remarkable potency, with MICs reduced by 4- to >128-fold (Figures 3A–3C; Figure S4). NP003 showed enhanced activity against specific *MCR-1*-bearing strains, and NP005 performed on par with colistin.

This superior activity extended to a diverse set of 10 clinical Gram-negative isolates with undefined resistance backgrounds. Against these strains, NP001, NP002, and NP004 consistently achieved lower MICs than colistin, whereas NP003 and NP005 displayed similar efficacy (Figure 3A; Figure S4).

To visually confirm these results and better model *in vivo* drug diffusion, we performed an antibiotic diffusion assay. As a representative example, NP001 produced clear, dose-dependent inhibition zones against *MCR-1*-expressing *E. coli* and *A. baumannii*, under conditions where colistin showed no detectable activity (Figures 3D and 3E). Collectively, these *in vitro* profiles establish NP001, NP002, and NP004 as promising candidates capable of overcoming prevalent polymyxin resistance. Given its consistently superior performance across all evaluations, we selected NP001 for further *in vivo* investigation.

### NP001 outperformed colistin in a murine thigh infection model

**Figure 4:**
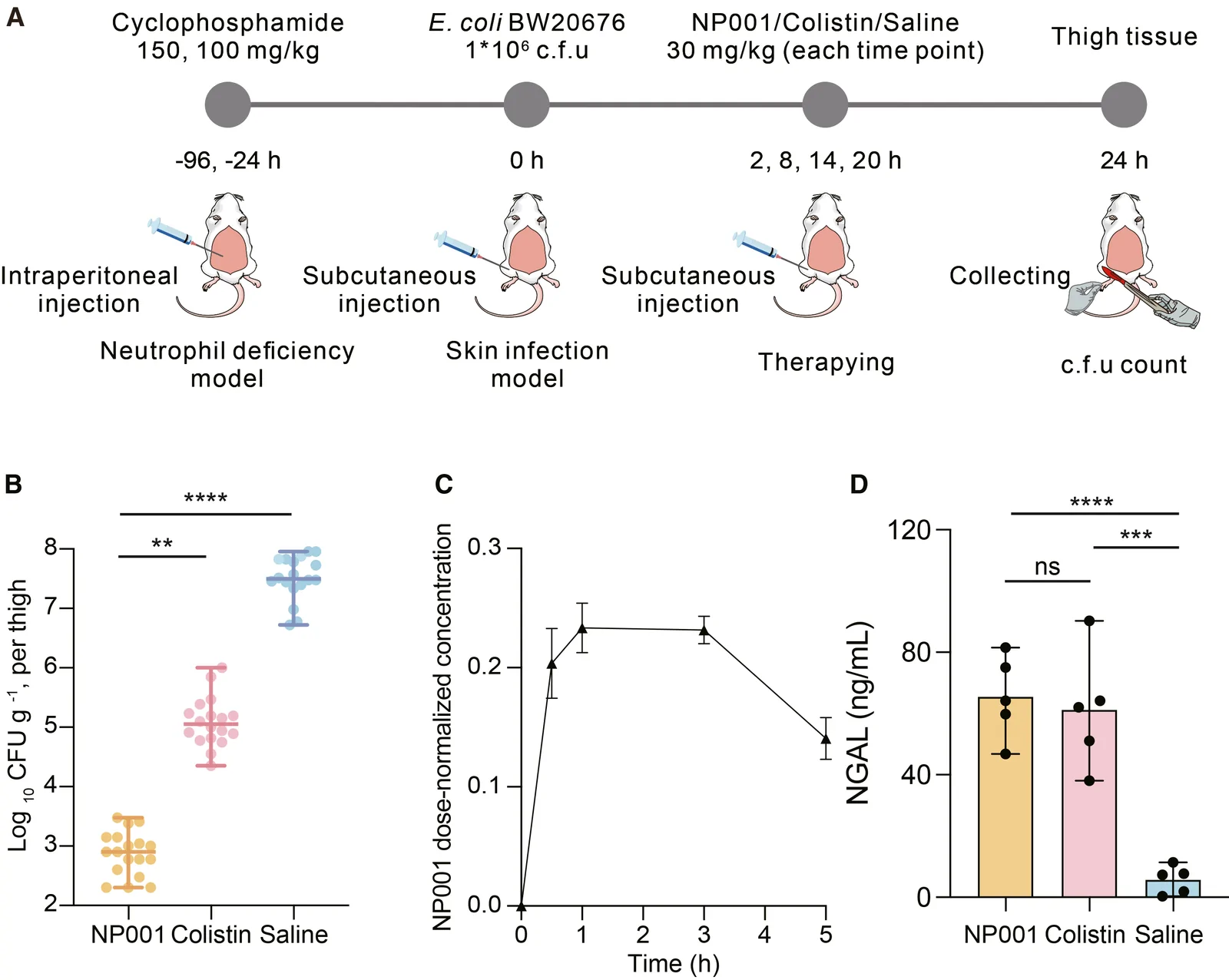
Therapeutic effects of NP001 on a mouse skin infection model (A) Experimental design of the NP001 treatment in the mouse skin infection model. (B) CFU count in mouse thigh tissue following NP001 treatment (*n* = 18, biological replicates). ∗∗*p* < 0.01, ∗∗∗∗*p* < 0.0001, unpaired *t* test. Error bars represent the median with range. (C) Metabolic profile of NP001 in mice following subcutaneous injection (*n* = 5, biological replicates). Error bars represent the SEM. (D) Plasma NGAL levels after subcutaneous injection of NP001, colistin, and saline (*n* = 5, biological replicates). ns, *p* > 0.05; ∗∗∗*p* < 0.001; ∗∗∗∗*p* < 0.0001, unpaired *t* test. Error bars represent the median with range.

To evaluate the *in vivo* efficacy of NP001, we established a murine thigh skin infection model, using a polymyxin-resistant *E. coli* strain, BW20676, expressing *MCR-1*. An initial dose-escalation study confirmed that the subcutaneous administration of NP001 at doses up to 40 mg/kg caused no mortality (Figure S5A). Based on this safety profile and considering the potential for increased drug susceptibility during active infection, a dose of 30 mg/kg was selected for subsequent efficacy evaluation. To further evaluate the *in vivo* safety of NP001, we statistically analyzed the body weight changes of mice treated with 30 mg/kg NP001. The results showed that, compared with the control group, both NP001- and colistin-treated groups exhibited reduced body weight, but no significant difference was observed between the NP001- and colistin-treated groups (Figure S5B). Concurrently, we performed H&E staining analysis on the mouse kidney tissues. The results indicated that, compared with the control saline group, neither NP001 treatment nor colistin treatment caused significant overall changes in the kidneys. Glomeruli appeared normal, with only mild acute tubular injury phenotypes observed, including slight tubular dilation, flattened and degenerated epithelial cells, and proteinaceous casts and cellular debris in some tubular lumens. Likewise, no significant differences were detected between the NP001 and colistin groups (Figure S5C). Behavioral observations showed that mice in the NP001-treated group maintained a higher level of spontaneous activity, whereas mice in the colistin-treated group exhibited reduced locomotor activity (Videos S1, S2, and S3). Together, these results indicate that both NP001 and colistin at 30 mg/kg are generally well tolerated in mice and can be used for subsequent efficacy studies in infection models. Furthermore, we quantified plasma neutrophil gelatinase-associated lipocalin (NGAL) after 7 days of repeated dosing.^13^ NGAL levels in the NP001-treated group were significantly elevated over the saline control but exhibited comparable toxicity to the currently clinically used colistin (Figure 4D). This indicated that the nephrotoxic potential of NP001 is comparable to that of the current standard-of-care therapy.

Video S1. Representative videos showing mouse activity after **subcutaneous** injection of NP001, related to Figure 4

Video S2. Representative videos showing mouse activity after **subcutaneous** injection of colistin, related to Figure 4

Video S3. Representative videos showing mouse activity after **subcutaneous** injection of saline, related to Figure 4

We then adopted an established protocol to assess the antimicrobial activity in immunocompromised mice. As outlined in Figure 4A, neutropenia was induced with cyclophosphamide prior to subcutaneous bacterial challenge. The mice were treated with NP001 (30 mg/kg), colistin, or saline at 2, 8, 14, and 20 h post-infection. Quantitative culture of thigh tissues harvested at the endpoint revealed that NP001 treatment reduced bacterial burdens by more than 100-fold compared with colistin treatment and by over 1,000-fold compared with the saline control treatment (Figure 4B), demonstrating superior antibacterial efficacy against a polymyxin-resistant pathogen.

Concurrent pharmacokinetic and safety assessments were conducted to profile NP001 for clinical translation. Following a single 30 mg/kg subcutaneous dose, NP001 rapidly achieved the peak plasma concentration within 1 h and maintained a sustained presence, with plasma levels at 5 h remaining at approximately 60% of the maximum (Figure 4C). This profile suggests a prolonged duration of action, which may be advantageous for managing persistent infections.

### NP001 confers a significant survival advantage in a lethal systemic infection model

**Figure 5:**
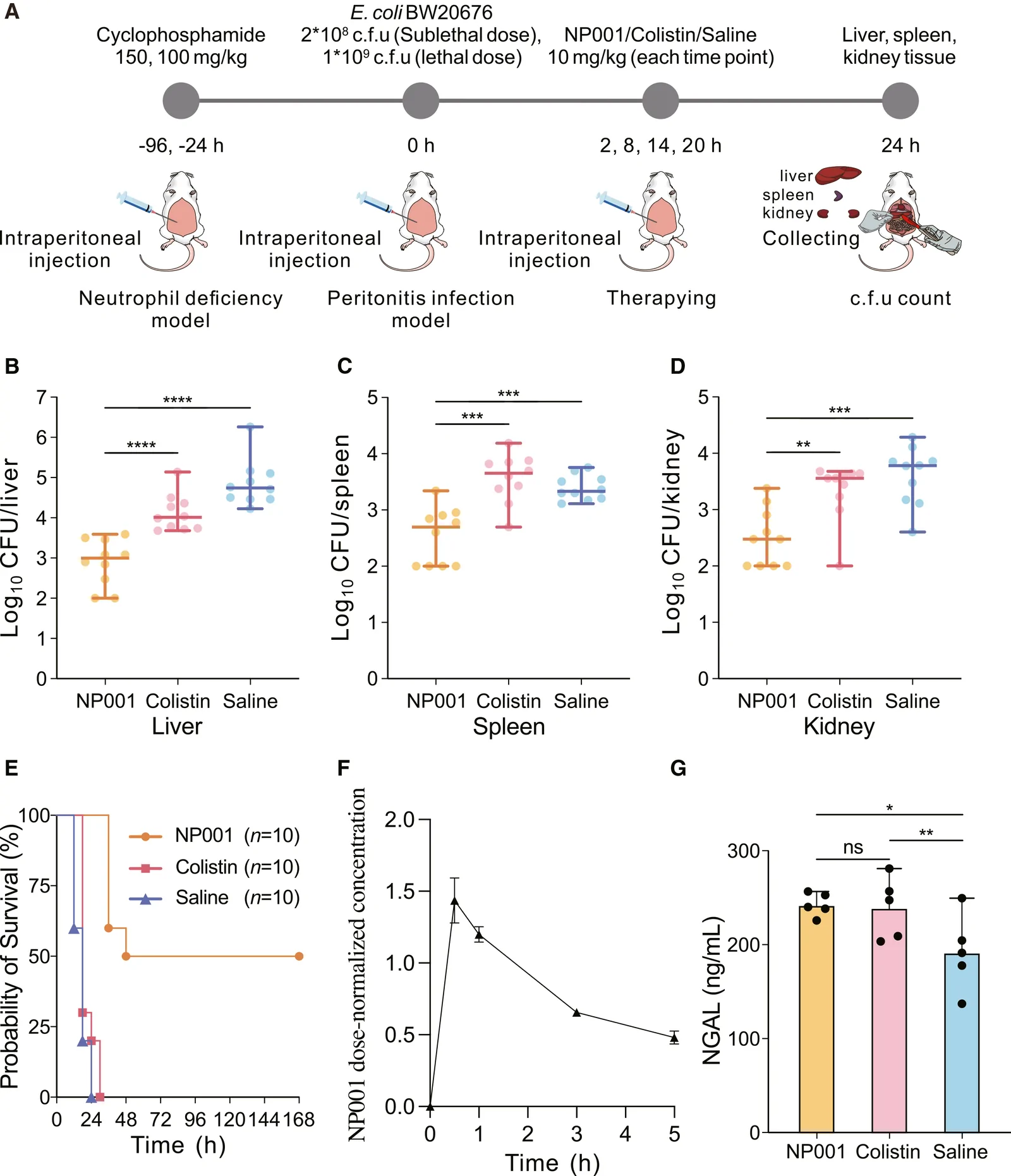
Therapeutic effects of NP001 in a mouse peritonitis infection model (A) Experimental design of NP001 treatment in the mouse intraperitoneal infection model. (B–D) CFU count in the liver (B), spleen (C), and kidney (D) of mice following antibiotic treatment (*n* = 10). ∗∗*p* < 0.01, ∗∗∗*p* < 0.001, ∗∗∗∗*p* < 0.0001, unpaired *t* test. Error bars represent the median with range. (E) Survival curve of mice under lethal-dose bacterial infection with antimicrobial peptide treatment (*n* = 10, biological replicates). (F) Metabolic profile of NP001 in mice following intraperitoneal injection (*n* = 5, biological replicates). Error bars represent the SEM. (G) Plasma NGAL levels after intraperitoneal injection of NP001, colistin, and saline (*n* = 5, biological replicates). ∗*p* < 0.05, ∗∗*p* < 0.01, unpaired *t* test. Error bars represent the median with range.

To assess the efficacy of NP001 against disseminated polymyxin-resistant infection, we established a murine intraperitoneal sepsis model. The mice were challenged with the *MCR-1*-expressing, polymyxin-resistant *E. coli* strain BW20676 (Figure 5A). A preliminary dose-ranging study determined that 1 × 10^9^ CFU constituted a lethal dose, causing 100% mortality within 24 h, while lower doses (≤1 × 10^8^ CFU) were non-lethal over 72 h (Figure S5D).

We first evaluated bacterial clearance at a sublethal challenge dose (2 × 10^8^ CFU) in immunocompromised mice. Treatment with NP001 (10 mg/kg) via intraperitoneal injection at 2, 8, 14, and 20 h post-infection resulted in a greater than 10-fold reduction in bacterial loads in the liver, spleen, and kidneys compared with colistin treatment, and an approximately 100-fold reduction compared with the saline control (Figures 5B–5D).

The therapeutic benefit of NP001 was most evident under a lethal challenge (1 × 10^9^ CFU). In this stringent model, all mice treated with colistin or saline succumbed to the infection within 36 h. In stark contrast, 50% of the NP001-treated mice survived for the entire 168-h observation period (Figure 5E), underscoring the potent, life-saving efficacy of NP001 against an otherwise fatal, polymyxin-resistant infection.

The pharmacokinetic profile of NP001 following intraperitoneal administration showed rapid absorption (Tmax = 0.5 h) and clearance, with plasma concentrations declining to near 30% by 5 h (Figure 5F). Consistent with observations from the thigh infection model, nephrotoxicity assessment revealed that the NGAL levels in the NP001-treated group were not significantly different from those in the colistin-treated group (Figure 5G), confirming a comparable renal safety profile at the efficacious dose.

### NP001 circumvents *MCR-1* resistance via direct membrane binding and disruption

The molecular mechanism by which AMPs overcome *MCR-1*-mediated polymyxin resistance remains poorly understood. Colistin exerts its bactericidal activity by targeting LPS: its positively charged diaminobutyric acid (Dab) residues form electrostatic interactions with the anionic phosphate groups of lipid A, while its fatty acyl chain inserts into the membrane via hydrophobic interactions (Figure S6A).^14^ The *MCR*-1-encoded phosphoethanolamine (PEA) transferase confers resistance by adding a PEA moiety to the lipid A phosphate group, neutralizing its charge and thereby abolishing the critical electrostatic attraction to colistin.

To elucidate how NP001 circumvents this resistance, we performed molecular docking simulations. Docking models suggest that NP001 may adopt a binding mode in which its Dab5, Dab8, and Dab9 residues are positioned near the polar groups of the glucosamine disaccharide region of lipid A, rather than relying primarily on interactions with phosphate groups (Figure S6B). This model provides a possible explanation for the retained activity of NP001 against *MCR*-1-expressing strains, that is, the binding of NP001 to lipid A may be less dependent on electrostatic interactions mediated by phosphate groups (Figure S6C).

**Figure 6:**
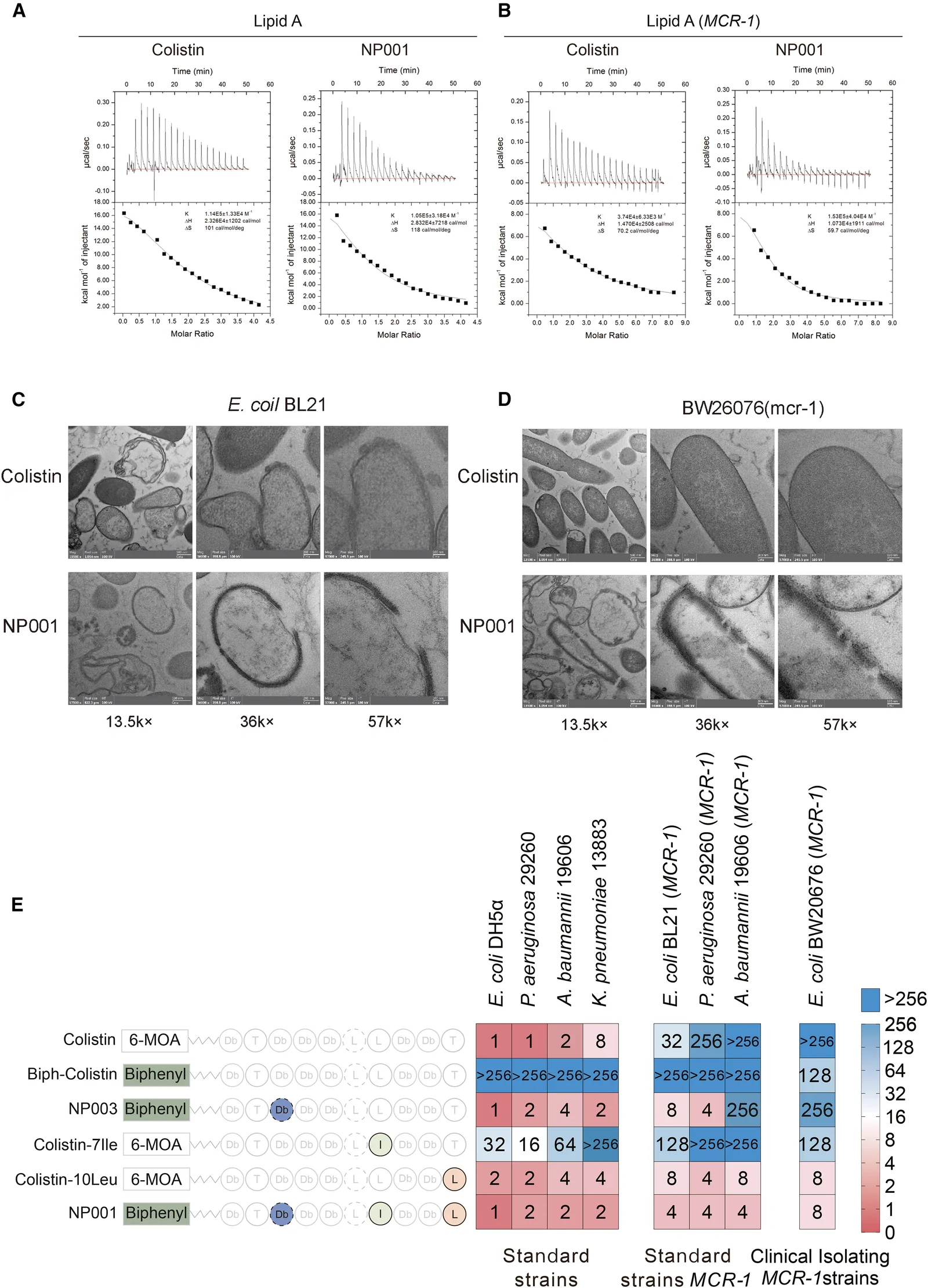
Analysis of the interactions between antimicrobial peptides and lipid A of the bacterial membrane and key amino acid residues underlying the ability of NP001 to overcome *MCR-1*-mediated resistance (A) Isothermal titration calorimetry (ITC) analysis of lipid A extracted from *E. coli* BL21 titrated with NP001 or colistin. Top: heat flow (μcal/s) versus time. Bottom: binding curves and fitted models for NP001 or colistin interacting with wild-type (WT) lipid A, generated using Origin 7.0. (B) ITC analysis of lipid A isolated from *E. coli* BW20676 (*MCR-1*) titrated with NP001 or colistin. (C) Morphological changes of the outer membrane of *E. coli* BL21 after treatment with NP001 or colistin. Representative images are shown at 13.5k×, 36k×, and 57k× magnifications. Scale bar, 500 nm, 200 nm, and 100 nm. (D) Morphological changes of the outer membrane of *E. coli* BW20676 (*MCR*-1) after treatment with NP001 or colistin. (E) MIC testing of multiple polymyxin variants. Left: amino acid sequences; right: MIC values of each peptide. All MIC assays were performed with three biological replicates.

Because the structures of peptides are highly flexible, the docking results can serve only as supporting evidence, and we further validated this mechanism experimentally using NP001. Isothermal titration calorimetry (ITC) confirmed the enhanced binding affinity of NP001 for MCR-1-modified lipid A (Figures 6A and 6B). The binding affinity of colistin to wild-type lipid A is 1.14 × 10⁵, while that of NP001 is 1.05 × 10⁵, with no significant difference between the two (Figures 6C and 6D). However, when the binding target is switched to *MCR*-*1*-modified lipid A, the binding ability of colistin decreases significantly, with a K value of 3.74 × 10^4^, whereas that of NP001 increases to 1.53 × 10⁵, demonstrating stronger binding affinity (Figures 6A and 6B). This result is also consistent with the docking data, revealing the binding pattern of NP001. Furtherly, transmission electron microscopy revealed that the treatment with NP001 caused severe outer membrane distortion and envelope contraction in *MCR-1*-expressing *E. coli*, phenotypes characteristic of lipid A-targeting agents (Figures 6C and 6D). In contrast, colistin treatment left the membranes of the resistant strain largely intact (Figures 6C and 6D). Crucially, this direct biophysical evidence demonstrates that our discovered peptides not only evade the neutralizing effect of *MCR-1* but actually achieve superior binding to the modified lipid A, leading to potent membrane disruption and bacterial killing.

### Residue 10 of NP001 is key to overcoming *MCR-1* resistance

Further, we sought to investigate the key structural changes that enable NP001 to overcome *MCR-1*-mediated resistance. Compared with colistin, NP001 possesses both an N-terminal fatty acyl chain and amino acid differences at positions 3 (D-Dab/Dab), 7 (Ile/Leu), and 10 (Leu/Thr). Therefore, we validated each of these differences one by one. We synthesized a colistin analog, Biph-Colistin, with only the N-terminal fatty acyl chain modified. Unexpectedly, in the *in vitro* MIC assays, Biph-Colistin failed to effectively inhibit the growth of laboratory standard strains or *MCR*-1-containing resistant strains (Figure 6E; Figure S4). Subsequently, we replaced the amino acids at the corresponding positions of colistin one by one with residues identical to those of NP001 and synthesized the resulting peptides. Among them, the position 3 substitution corresponds to NP003 constructed previously, and the position 7 and position 10 variants were named Colistin-7Ile and Colistin-10Leu, respectively. MIC results showed that Colistin-7Ile exhibited a substantial decrease in antibacterial activity; NP003 maintained inhibition of the standard strain but significantly inhibited two of the *MCR*-1-containing bacterial strains while having no notable effect on the other two; Colistin-10Leu displayed significant inhibitory activity against all tested strains, very close to that of NP001 (Figure 6E; Figure S4). These results indicated that the substitution of threonine with leucine at position 10 is one of the key factors enabling NP001 to overcome *MCR*-1-mediated resistance, while other positions such as 3 and 7 may further enhance the overall antibacterial effect.

## Discussion

Current NRPS mining tools include antiSMASH, Stachelhaus code, NRPSpredictor2, SANDPUMA, and AdenPredictor, among others. These tools are primarily used under the premise that NRPS BGCs and modules have been identified and that the module order is largely clear to predict the amino acid type corresponding to each module based on A domain features. Similarly, existing synthetic-bioinformatic natural product (syn-BNP) studies, although able to predict candidate peptides from incomplete BGCs, still typically rely on resolvable core NRPS module information to deduce the peptide backbone; the incompleteness is more often reflected in the absence of tailoring enzymes, insufficient expression information, or the inability to directly obtain natural products, rather than necessarily addressing the issue of “how to determine amino acid positions when the module order is unclear.” In contrast, the key challenge faced by this study is that in many incomplete or fragmented BGCs, relying solely on A domains enables only determination of the candidate amino acid types but cannot help in reliably establishing the positions of these amino acids in the final peptide. Therefore, this study further introduces branching information from phylogenetic trees of the C and T domains to assist in inferring AA positions, and combines this with AA types inferred from the A domain phylogeny to reconstruct candidate peptide sequences. We first systematically constructed the algorithm, using polymyxin analogues as an example.

By decoding the evolutionary signatures embedded in individual A, C, and T domains, we successfully mined the vast and underexplored reservoir of fragmented genomic data from 954 *Paenibacillus* genomes. This approach led to the identification of five unreported polymyxin analogues, with NP001 emerging as a potent candidate capable of overcoming *MCR-1*-mediated resistance both *in vitro* and in murine infection models.

A pivotal finding of our study is that the C and T domains of the NRPS machinery carry phylogenetic signals that are strongly correlated with the positional assignment of their corresponding amino acids within the final peptide scaffold. From a molecular evolution perspective, the A domain selectively recognizes different amino acid substrates based on structural differences, serving as the key determinant in the substrate selection for synthesis. Given that the amino acid sequence of the A domain dictates its structure, it is not surprising that the substrate specificity can be predicted from sequence data. Interestingly, despite their distinct roles—with C domains facilitating amino acid ligation and T domains enabling polypeptide elongation—both domains exhibit functional conservation while also displaying clustering patterns related to the position of the encoded amino acid. Looking at the predicted protein structures of the C/T domain, we did not find significant differences (Figures S7A and S7B). Then, it is possible that the clustering of its sequence differences leading to the association with the coding amino acid positions is a result of the hitchhiking effect after domain duplication during the evolutionary process. This may be related to the evolutionary history of this gene family and deserves further exploration to uncover the underlying mechanisms (Figures S7A and S7B).

Is the evolution-based strategy for mining AMPs similarly applicable to other NRPSs? We further illustrate this by using bacitracin as an example. Bacitracin is a naturally occurring AMP produced by *Bacillus* species, primarily *Bacillus licheniformis* and *Bacillus subtilis*. Bacitracin inhibits cell wall synthesis by blocking the recycling of the cell wall precursor lipid carrier, thereby exhibiting good antibacterial activity against Gram-positive bacteria. It is now widely used in clinical anti-infection therapy and animal husbandry.^15^ Bacitracin is also synthesized in a modular strategy by using NRPSs; therefore, its biosynthesis likewise relies on functional domains such as A, C, and T. Similar to polymyxins, when the sequencing data contain incomplete NRPS BGCs, existing algorithms struggle to directly deduce the amino acid composition and positional information of the peptide. Using genomic data from 41 *Bacillus* species, we followed the workflow shown in Figure 1E to dissect the NRPS domains within the bacitracin BGC and constructed phylogenetic relationships linking the A, C, and T domains to their corresponding amino acid types and positions (Table S5). The results showed that, in *bacillus*, the A domains also cluster according to the amino acids they encode (Figure S2A), whereas the C and T domains cluster according to the positions of the encoded amino acids (Figures S2B and S2C). Therefore, we believe that the evolution-based peptide mining strategy presented in this study has broad applicability.

Meanwhile, we further compared the similarities and differences between the AMPs detected using the antiSMASH method and the algorithm developed in this study. In the *Paenibacillus* genus dataset, antiSMASH could predict a total of 98 complete peptides, which fell to 10 after deduplication; of these 10 peptides, three were previously unreported (NP001–003 identified in this study) (Table 1; Table S3).Notably, this dataset was generated using the standard antiSMASH prediction criteria and therefore differed from the more stringent reference dataset (35 BGCs) used for phylogenetic model construction. However, our strategy additionally identified two unreported AMPs, NP004 and NP005, of which NP004 effectively inhibited the growth of polymyxin-resistant strains, demonstrating a stronger ability to discover more candidates. On the other hand, recent studies have discovered thousands of candidate AMPs through AI-assisted mining or structure prediction, but their actual antimicrobial activity rarely matches that of natural AMPs, and those capable of overcoming drug resistance are even rarer. For example, Torres et al. (2024) predicted 323 candidate AMPs from small open reading frames of the human and microbiome origin, yet after synthesis and validation, none exhibited broad-spectrum antimicrobial activity; only 4 peptides (MIC ≤ 4 μM) showed colistin-comparable activity against specific bacteria, and their ability to inhibit *MCR-1*-resistant strains was not further validated.^7^ In contrast, our approach directly performs targeted mining from polymyxin-producing bacteria. Because these strains have long been engaged in a natural antibiotic “arms race,” their secondary metabolites are more likely to possess genuine antimicrobial activity and harbor adaptive evolution geared toward resistance mechanisms. Hence, our algorithm not only achieves a higher hit rate for candidate identification but also facilitates the discovery of entirely new AMPs that are active against clinically resistant bacteria.

**Table 1:** Comparison of products identified by antiSMASH and our algorithm

	antiSMASH	Evolution-inspired algorithm	Reported
Polymyxin A	yes	yes	yes
Polymyxin B	yes	yes	yes
Polymyxin B-Dab3	yes	yes	yes
Polymyxin D	yes	yes	yes
Polymyxin E	yes	yes	yes
Polymyxin P	yes	yes	yes
Macolacin	yes	yes	yes
Polymyxin B-Ile	no	yes	yes
Polymyxin E-Ile	no	yes	yes
Polymyxin M	no	yes	yes
Polymyxin S	no	yes	yes
Polymyxin T	no	yes	yes
NP001	yes	yes	no
NP002	yes	yes	no
NP003	yes	yes	no
NP004	no	yes	no
NP005	no	yes	no

Our mechanistic studies reveal that NP001’s success stems from a fundamental shift in its molecular target on lipid A. ITC experiments provided quantitative binding evidence for the interaction between NP001 and membrane-associated targets; electron microscopy observations revealed obvious membrane damage and lysis phenotypes in bacteria following NP001 treatment; and further amino acid substitution experiments indicated that the residue at position 10 plays a critical role in the antibacterial activity of NP001 (Figures 6A–6E). Together, these results support a membrane-targeting mode of action for NP001 at three levels (biophysical binding, cellular morphology, and structure-activity relationships). Given the flexible nature of peptides, docking serves only as a complementary approach, supporting the binding mode of the AMP to the cell membrane. This alternative binding mode, combined with its superior binding affinity for modified lipid A, allows it to effectively disrupt membranes and kill polymyxin-resistant bacteria. This mode of action, which is highly dependent on membrane-binding conformation, also helps explain why seemingly minor changes in sequence or stereochemistry can lead to significant differences in activity. NP003 differs from Biph-Colistin only in the stereochemistry of the Dab residue at position 3, yet the two compounds exhibit markedly different antibacterial activities, with the magnitude of change being greater than that typically observed for Dab stereochemical alterations in previous polymyxin activity studies. Similarly, the Leu-to-Ile substitution at position 7 of colistin results in 16-fold or higher MIC changes across multiple strains, although some previous studies have generally considered this conservative hydrophobic substitution to have a minor impact on polymyxin activity. These results indicate that the activity of polymyxin-class peptides does not depend solely on the amino acid type, net charge, or hydrophobicity, but rather on the three-dimensional synergy among the N-terminal acyl chain, the N-terminal linear peptide segment, the cyclic peptide core, the distribution of positive charges on the Dab side chains, and the membrane-binding hydrophobic surface. Although the D-/L-Dab change does not alter the net charge, it may alter the local backbone conformation, the projection direction of the Dab side chain, and the spatial orientation of the N-terminal biphenyl acyl chain relative to lipid A, thereby significantly affecting membrane binding and antibacterial activity; Such results, in which a single amino acid substitution leads to pronounced changes in MIC, have also been reported repeatedly in previous studies.^16^^,^^17^^,^^18^^,^^19^ Therefore, such few site changes may lead to loss, retention, or restoration of activity in different peptide backbone contexts, further demonstrating that identifying naturally conserved sequence combinations from incomplete BGCs based on evolutionary information can help uncover anti-resistance activity combinations that are difficult to predict using empirical single-point mutagenesis or conventional rational design.

Meanwhile, the influence of the N-terminal acyl chain on the activity of polymyxin-class peptides cannot be simply generalized. Previously, Wang et al. found in macolacin that replacing the native 6-MOA acyl chain with a biphenyl acyl chain could significantly enhance its activity against polymyxin-resistant bacteria.^4^ However, in the present study, the biphenyl acyl chain exerted differential effects on different peptide backbones. Biph-NP001 and 6-MOA-NP001 exhibited largely comparable MIC values across the tested strains (Figure S6D), indicating that the antibacterial activity of NP001 does not depend on the biphenyl acyl chain per se. In contrast, when colistin was converted from 6-MOA to the biphenyl analogue, its antibacterial activity decreased markedly, with MIC values rising by dozens of folds. These results suggest that the N-terminal acyl chain is not an independent module that dictates activity on its own; the same biphenyl acyl substitution can lead to enhanced activity, essentially unchanged activity, or diminished activity across different polymyxin-like scaffolds, such as macolacin, NP001, and colistin. Therefore, the activity of polymyxin-class molecules arises from the overall structural compatibility between the N-terminal acyl chain and the peptide core, rather than from a linear contribution of any single structural moiety.

Beyond the specific discovery of NP001, our work establishes a generalizable framework for NRP discovery. The evolutionary imprint deciphered from the C and T domains—now deciphered—enables the functional interpretation of orphan domains. The dataset of 1,981 characterized domains from the polymyxin pathway we provide serves as a blueprint for the synthetic biology-driven design of custom NRPs, paving the way for the *de novo* synthesis of tailored therapeutic peptides.

Looking forward, our data clearly indicate opportunities for clinical optimization. The tolerability of higher doses (up to 40 mg/kg) and the pharmacokinetic profile of NP001 suggest that both dose escalation and interval adjustment could further enhance efficacy. Moreover, advanced delivery strategies, such as the nanoparticle encapsulation already demonstrated for polymyxin B, could be leveraged to maximize target-site concentration and minimize systemic exposure, potentially achieving an even more favorable therapeutic index.^20^

### Limitations of the study

Although the present study reveals certain phylogenetic relationships among the A, C, and T domains within NRPS BGCs that can be used to predict amino acid type and position information, the underlying reason why the sequences of the C and T domains carry the corresponding positional information of the peptide remains unresolved. Elucidating the molecular mechanism in the future would facilitate targeted engineering of specific NRPS domains in synthetic biology, thereby advancing the *de novo* design and synthesis of natural products based on BGCs.

In addition, although our results support that NP001 overcomes *MCR-1*-mediated resistance through interaction with lipid A and identify Leu10 as a critical residue contributing to this phenotype, the atomic-level binding mechanism remains unclear. Structural studies, such as NMR spectroscopy or co-crystallization of NP001 with lipid A, may further reveal the molecular basis underlying this interaction. Moreover, the mechanisms of polymyxin resistance are diverse, including *MCR-1* and other lipid A modification pathways. This study primarily focuses on the inhibitory activity of NP001 against *MCR-1*-mediated resistant bacteria, and its applicability to other polymyxin resistance mechanisms requires further investigation. On the other hand, NP001 exhibits toxicity comparable to that of colistin; therefore, exploring whether broader chemical modifications can further reduce its toxicity is an important step for clinical translation. Finally, the *in vivo* safety evaluation in this study is mainly based on short-term administration models. The long-term toxicity, tissue accumulation profile, and systemic pharmacokinetic characteristics of NP001 remain to be further characterized.

## Resource availability

### Lead contact

Requests for further information and resources should be directed to the lead contact, Qihan Chen (lyonchen@umac.mo).

### Materials availability

All peptides generated in this study are available from the lead contact upon request, subject to completion of a material(s) transfer agreement (MTA).

### Data and code availability

• This paper analyzes existing, publicly available bacterial genome assemblies downloaded from NCBI GenBank/RefSeq. All data reported in this paper will be shared by the lead contact upon request.
• This paper analyzes existing, publicly available *Bacillus* and *Paenibacillus* genome assemblies downloaded from NCBI. The accession numbers of the genome assemblies analyzed in this study are provided in Table S5. Original code and custom scripts for the A/C/T-domain-informed phylogenetic reconstruction workflow have been deposited at GitHub and are publicly available as of the date of publication at https://github.com/Lexie1229/Evolutionary-Inspired-Antimicrobial-Peptides-Identification.
• Any additional information required to reanalyze the data reported in this paper is available from the lead contact upon request.

## Acknowledgments

We are grateful to the High-Performance Computing Center of Nanjing University for doing the numerical calculations in this paper on its blade cluster system. We thank Prof. Hui Wang from the College of Life Sciences, Nanjing Agricultural University, for providing the *E. Coli* BW20676 (*MCR*-1) strain, and Prof. Beiwen Zheng from the First Affiliated Hospital, Zhejiang University School of Medicine, for providing the remaining clinical strains. This work was supported by the Science and Technology Innovation Key R&D Program of Chongqing (CSTB2024TIAD-STX0043), Science and Technology Development Fund, Macao S.A.R (10.13039/501100006469FDCT) (0102/2024/AFJ) and (0001/2024/RIC), and Jiangsu Funding Program for Excellent Postdoctoral Talent (2024ZB720).

## Author contributions

Conceptualization, Q.C., Q.W., J.G, and J.-Q.C.; methodology, Q.W. and J.G.; investigation, Y.T., X.C., Jing Zhou, Y.S., and Z.C; visualization, L.Z., Y.T., Jing Zhou, and Jiaying Zhou; validation, Y.T., X.C., Jing Zhou, Y.S., and Z.C.; writing – original draft, Y.T.; writing – review & editing, Q.C., Q.W., J.G., L.Z., and Y.T.; supervision, Q.C. and Q.W.; resources, J.-Q.C.; provision of bacterial strains, Y.Z.

## Declaration of interests

A patent has been filed relating to the data presented.

## STAR★Methods

### Key resources table

**Table:** 

REAGENT or RESOURCE	SOURCE	IDENTIFIER
**Bacterial and virus strains**		

*E. coli* DH5a	Vazyme Biotech Co., Ltd.	Cat#C502
*P. aeruginosa* 29260	ATCC	Cat# ATCC® 29260™
*A. baumannii* 19606	Beijing BioBW Biotechnology Co., Ltd.	Cat# bio-53250
*K. pneumoniae* 13883	ATCC	ATCC® 13883™
*E. coli* BL21 (MCR-1)	this study	N/A
*P. aeruginosa* 29260 (MCR-1)	this study	N/A
*A. baumannii* 19606 (MCR-1)	this study	N/A
*E. coli* BW20676 (MCR-1)	Kindly provided by Prof. Hui Wang (College of Life Sciences, Nanjing Agricultural University)	N/A
*K. pneumoniae* 19333	Nanjing Drum Tower Hospital	N/A
*K. pneumoniae* 19337	Nanjing Drum Tower Hospital	N/A
*P. aeruginosa* 3133K	Kindly provided by Prof. Beiwen Zheng (First Affiliated Hospital, Zhejiang University School of Medicine)	N/A
*P. aeruginosa* 7118K	Kindly provided by Prof. Beiwen Zheng (First Affiliated Hospital, Zhejiang University School of Medicine)	N/A
*P. aeruginosa* 42507	Kindly provided by Prof. Beiwen Zheng (First Affiliated Hospital, Zhejiang University School of Medicine)	N/A
*P. aeruginosa* 42059	Kindly provided by Prof. Beiwen Zheng (First Affiliated Hospital, Zhejiang University School of Medicine)	N/A
*P. aeruginosa* 42720	Kindly provided by Prof. Beiwen Zheng (First Affiliated Hospital, Zhejiang University School of Medicine)	N/A
*P. aeruginosa* E55K	Kindly provided by Prof. Beiwen Zheng (First Affiliated Hospital, Zhejiang University School of Medicine)	N/A
*P. aeruginosa* 44253	Kindly provided by Prof. Beiwen Zheng (First Affiliated Hospital, Zhejiang University School of Medicine)	N/A

**Chemicals, peptides, and recombinant proteins**		

NP001	this study	N/A
NP002	this study	N/A
NP003	this study	N/A
NP004	this study	N/A
NP005	this study	N/A
Biph-Colistin	this study	N/A
Colistin_7Ile	this study	N/A
Colistin_10Leu	this study	N/A
6-MOA-NP001	this study	N/A

**Critical commercial assays**		

Mouse NGAL/Lipocalin-2 ELISA Kit	Beyotime Biotechnology	Cat#PN757
H&E staining kit	BIOSSCI Biotech Co., Ltd	Cat#BP0211
TRYPTONE	Thermo Scientific Oxoid	Cat#LP0042
Yeast Extract	Thermo Scientific Oxoid	Cat#LP0021
NaCl	Bide Pharmatech Co., Ltd.	Cat#BD01122789
Colistin sulfate	TargetMol Chemicals Inc.	Cat#T1245
Antibiotic Disk(blank)	Liofilchem S.r.l.	Cat#9999/102723092
Cyclophosphamide monohydrate	BioRuler	Cat#RH31543
DMSO	Shanghai Acmec Biochemical Technology Co., Ltd.	Cat#D54265
4% paraformaldehyde	Biosharp Life Sciences	Cat#BL53 9A
Methanol	Shanghai Aladdin Biochemical Technology Co., Ltd.	Cat#67-56-1
Isopropyl-beta-D-thiogalactopyranoside	Beijing Solarbio Science & Technology Co., Ltd.	Cat#I8070
Glutaraldehyde	Shanghai Aladdin Biochemical Technology Co., Ltd.	Cat#G359127
K4Fe(CN)6	Shanghai Macklin Biochemical Co., Ltd.	Cat#P816420
ClonExpress Ultra One Step Cloning Kit	Vazyme Biotech Co., Ltd.	Cat#C115
2 × Phanta Max Master Mix (Dye Plus)	Vazyme Biotech Co., Ltd.	Cat#P525

**Deposited data**		

Publicly available bacterial genome	NCBI	Accession number are provided in Table S5

**Experimental models: Organisms/strains**		

BALB/c	Vital River Laboratory Animal Technology Co., Ltd.	N/A

**Oligonucleotides**		

Primer: MCS-plasmid-F	Sangon Biotech	Table S4
Primer: MCS-plasmid-R	Sangon Biotech	Table S4
Primer: MCS-MCR-F	Sangon Biotech	Table S4
Primer: MCS-MCR-R	Sangon Biotech	Table S4

**Recombinant DNA**		

MCR-1 gene fragment	GenScript Biotech Co., Ltd.	Available from the lead contact upon request

**Software and algorithms**		

antiSMASH	v7.0.0	https://github.com/antismash/antismash
nwr	v0.7.0	https://github.com/wang-q/nwr
MAFFT	v7.520	https://mafft.cbrc.jp/alignment/software/
trimAl	v1.4.rev15	https://github.com/inab/trimal
FastTree	v2.1.11	http://www.microbesonline.org/fasttree/
table2itol.R	v2.12	https://github.com/mgoeker/table2itol
iTOL	v6	https://itol.embl.de/
CD-HIT	v4.8.1	https://github.com/weizhongli/cdhit
Easel	v0.46	https://github.com/EddyRivasLab/easel
HMMER	v3.4	https://github.com/EddyRivasLab/hmmer
miniprot	v0.13-r248	https://github.com/lh3/miniprot
BLAST	v2.15.0+	https://blast.ncbi.nlm.nih.gov/
faops	v0.8.21	https://github.com/wang-q/faops
AlphaFold3	v3.0.0	https://github.com/google-deepmind/alphafold3
USalign	Version 20241108	https://github.com/pylelab/USalign
PyMOL	v2.5.0	https://github.com/schrodinger/pymol-open-source
ChemOffice	v22	https://revvitysignals.com/products/research/chemdraw
Gaussian 16	Gaussian 16	https://gaussian.com/
A/C/T-domain-informed phylogenetic workflow for incomplete BGC mining	This paper	https://github.com/Lexie1229/Evolutionary-Inspired-Antimicrobial-Peptides-Identification
Prism (v9.3.1 or newer)	GraphPad	https://www.graphpad.com

### Experimental model and study participant details

#### Bacterial strains and genomes

To analyze domain-specific patterns in BGCs, we first retrieved the genomes of 954 *Paenibacillus* genomes from the NCBI database (as of August 8, 2023). To ensure the reliability of phylogenetic relationship construction, we screened genomes that contained BGC information and their associated products using antiSMASH for domain-specific pattern exploration. Following the standard antiSMASH (v7.0.0) workflow, we identified 35 complete BGCs along with their corresponding peptide products.

For the identification of bacitracin BGCs in the genus *Bacillus*, all 41 species across 7 families in the NCBI RefSeq database (as of November 5, 2024) were downloaded. The entire process was completely consistent with the identification of polymyxin analogue BGCs in the genus *Paenibacillus*.

The MCR-1 gene fragment (NCBI accession: CP100017.1, 23338–24963 bp region) was synthesized and cloned into the pET28a(+) plasmid by GenScript Biotech Ltd. The recombinant plasmid was then transformed into *E. coli* BL21, and *MCR-1* protein expression was induced by adding IPTG to a final concentration of 20 μm.

Additionally, the MCR-1 gene was inserted into the pBBR1MCS2 plasmid via homologous recombination, and Sanger sequencing was performed to verify sequence integrity. Then, electrocompetent cells of *P. aeruginosa* 29260 and *A. baumannii* 19606 were prepared. The transformed products were subsequently plated onto selective agar plates containing polymyxin (20 μg/mL) and incubated at 37°C for 16 h. Single colonies were picked and verified by MIC assays before being stored for further use.

*E. coli* BW20676 (MCR-1) and *E. coli* BL21 strains were cultured in LB broth at 37°C. For lipid A extraction, strains were cultured until OD600 reached approximately 1.2. For transmission electron microscopy, bacterial suspensions were adjusted to a concentration of 1 × 10^8^ CFU/mL and incubated with 5 μg/mL of NP001 or colistin E for 8 h.

#### Mouse models

All mouse experiments were conducted in accordance with ethical guidelines and approved by the Ethics Committee of Army Medical University (Ethics ID: AMUWEC20223336). To establish a neutropenic mouse model, six-week-old BALB/c female mice (18–22 g) were used in this experiment. Mice were randomly housed in individually ventilated cages and maintained according to the standards of the American Association for Laboratory Animal Science (AALAS). The housing environment was controlled with a 12-h light/dark cycle, a temperature of 21°C, and a humidity of 30%. To induce neutropenia, mice were injected intraperitoneally with cyclophosphamide at doses of 150 mg/kg on day 4 and 100 mg/kg on day 1 prior to bacterial infection.

Six-week-old BALB/c female mice (18–22 g) were used for nephrotoxicity assessment. The mice were housed under controlled conditions, with a 12-h light/dark cycle, a temperature of 21°C, and a humidity of 30%. After 3 days of acclimatization, mice were randomly divided into three groups (*n* = 5 per group): the carrier (0.9% saline) group, the Colistin group, and the NP001 group.

Six-week-old BALB/c female mice (18–22 g) were used for pharmacokinetic assays. The mice were housed under controlled conditions, including a 12-h light/dark cycle, a temperature of 21°C, and a humidity of 30%. Following 3 days of acclimatization, pharmacokinetic experiments were conducted.

### Method details

#### Peptide identification workflow

The data screening process consists of three key stages: Exploring domain-specific patterns and relationships using a dataset with characterized product associations; Establishing a classification framework based on these patterns for systematic evaluation of new data; Screening the dataset for potential antimicrobial peptides based on the established criteria.

##### Exploration of domain patterns in BGCs

We used nwr (v0.7.0) to download bacterial genomes of the genus *Paenibacillus* from the NCBI RefSeq database. As of 2023.08.08, a total of 954 bacterial genomes of the genus *Paenibacillus* were downloaded from the database, and the fna file of each genome was used as an input file for subsequent sequence searches.

Next, representative BGCs were identified for phylogenetic analysis. AntiSMASH (v7.0.0) was used to identify the biosynthetic gene clusters (BGCs) of secondary metabolites in all downloaded bacterial genomes of the genus *Paenibacillus* according to a standardized procedure, and obtained a total of 35 polymyxin BGCs that were complete and synthesized with well-defined peptide products.

We split and extracted the amino acid sequences of the A, C, and T domains of 10 modules of the 35 BGCs, and their corresponding amino acid types or position information, respectively. MAFFT (v7.520) was used for multiple sequence alignment, trimAl (v1.4. rev15) for sequence trimming, and FastTree (v2.1.11) for constructing phylogenetic trees in sequence, respectively. Subsequently, nwr (v0.7.0) was used to sort the unrooted phylogenetic trees in ascending order based on the number of descendants of each node, and table2itol.R (v2.12) to generate annotation files based on the attribute information of the functional classifications and the positional information, and finally, the online website iTOL (v6) was used to sort the sorted unrooted trees and their annotation files were visualized.

##### Establishment of reference sequences for amino acid types and positional information of polypeptides encoded by A, C and T domains

Based on the phylogenetic analysis of A, C and T domain, it is known that the clustering situation of A domain directly corresponds to the recognized amino acid type, and the clustering of C and T domain directly corresponds to the position where the module is located. Therefore, we can construct the model of A domain with the recognized amino acid types and the model of C and T domain with the position information of the module where it is located to resolve the product or position information corresponding to the unknown domain. A domain was classified into 8 categories, such as A_Dab, A_Leu, based on its ability to recognize 8 amino acids. There are 10 C and T domains in each BGC, which will be classified into 10 types, such as C_1, T_1, etc., respectively, and a total of 28 categories were obtained. Subsequently, the representative sequences of each category were filtered as models for subsequent identification of domains to be mined. The following is the detailed model construction process.

##### Filtering representative sequences for identifying the sequence composition of A, C and T domains

In order to find out which BGCs exist in unknown sequencing files, we first need to specify the representative sequences for identifying A, C and T domains. Cd-hit (v4.8.1) was used to cluster the amino acid sequences of the A, C, and T domains in each of the 35 BGCs. In order to ensure the premise that different classes of sequences from the same domain are distinguished as much as possible while retaining fewer representative sequences, we determined the sequence identity threshold for clustering based on the similarity of individual domains, where the identity thresholds for the A/C/T domains were 0.95/0.96/0.94, and finally 50/61/37 sequences were screened and obtained as representative sequences for identifying A, C and T domains, respectively.

##### Constructing models of A, C and T domains

The representative amino acid sequences will be screened out to be used to construct the models of each category. We take the clustering results of cd-hit (v4.8.1) in (1) and then remove the duplicate amino acid sequences. In order to retain more sequence types, the homogeneity thresholds were all set to 1.0. The sequences were then normalized and converted to the required format using esl-reformat (Easel v0.46). Profile HMM files were constructed for each category using hmmbuild (HMMER v3.4) with representative amino acid sequences, and the resulting profile HMMs were used as models for subsequent domain type determination.

##### Identification of peptides in the dataset for screening. Determination of BGC locations and nucleotide sequences

Representative sequences of A, C, and T domains as query sequences to perform sequence searches among the to-be-mined sequences using miniprot (v0.13-r248) and tblastn (BLAST v2.15.0+), respectively. The score threshold was set to 0.85 (--outs, output if score at least FLOAT∗bestScore) when miniprot was used to sequence search, as well as when tblastn sequence search was used, a local database was constructed for the genome first, and then performed sequence search on the genome. After that, python script was used to batch process the matches from miniprot sequence search (the organizing process includes removing outliers, and then merging different matches at the same location), and python script was used to batch process the matches from tblastn sequence search (the organizing process includes removing outliers, matches with lower confidence, and matches with lower quality), and then merge the different matches with overlap ≥0.95 between miniprot and tblastn positions. Based on a specific range of distances between A, C, and T domains determined from the statistics of the complete polymyxin BGC, the search results of representative sequences in the genome, i.e., domains , was divided into clusters. Each domain of each cluster in each genome was treated as a match, and its position information was extracted separately, and the nucleic acid sequence corresponding to each match was extracted from the genome using faops (v0.8.21).

##### Identification of A, C, and T Domains types

Perl scripts were used to obtain protein sequences by six-frame translation of each matched nucleic acid sequence. Subsequently, the profile HMMs files were used with different categories of A, C, and T domains as models, and performed sequence searches for each matched protein sequence using hmmscan (HMMER v3.4), we determined the model category matched by each matched protein sequence, and filtered the A, C, and T domains according to their respective thresholds for e-value and score sequence search results. In this case, the thresholds of e-value and score were obtained in the following way: we took each matched protein sequence of the genome where antiSMASH was able to identify the complete Polymyxin BGC, and counted the matches after searching with hmmscan using the profile HMMs files of the different classes of the A, C, and T domains as the models, the match value is the threshold value here.

##### Organize the final identification results

To organize the BGC identification results, we first remove the clusters that contain only 1 domain or do not contain A domain in each genome, and then organize the domain categories of the final identification of each match one by one, so as to ultimately determine the BGC situation of each cluster in each genome. In particular, for a match where multiple possible domain categories exist, we constructed a phylogenetic tree of the protein sequence of this match with all the representative sequences of its domain type, and based on the results of the phylogenetic analysis, we determined the most likely predicted category for this match. For example, the amino acid at site 6 in the vast majority of strains would be predicted to be D-Leu or D-Phe. We would further perform a phylogenetic analysis of the sequence of this A domain with the sequences of all A domains, and observe that it clusters with the A domain branch that identifies whether D-Leu or D-Phe is present, thus determining the specific amino acid site. If the product contains 10 amino acids and the corresponding position information, it is considered as a complete polypeptide, otherwise it is made up as a complete polypeptide based on the type of amino acid that has been previously reported for the missing site. For example, 10 complete modules could be identified in strain P_barci_GCF_013347305_1, and we directly integrated them to obtain the complete decapeptide molecule NP001 based on the corresponding sequences and positions of each of its modules; or for example, the 9th module was not identified in strain P_assam_DSM_18201_GCF_000422445_1 and the reported amino acid type at this site is L-Dab, so this amino acid was used as a complement to obtain the complete decapeptide molecule NP004.

For the identification of bacitracin BGCs in the genus *Bacillus*, all 41 species genome across 7 families in the NCBI RefSeq database (as of November 5, 2024) were downloaded. The entire process was completely consistent with the identification of polymyxin analogs BGCs in the genus *Bacillus*.

##### Peptide synthesis and purification

The synthesis, purification and identification of antimicrobial peptides were illustrated using NP001 as an example.

Dichloromethane (10 mL/g) was added to Fmoc-Leu-CTC resin and allowed to soak for 30 min, ensuring complete solubilization. After solvent removal, 20% piperidine in DMF (10 mL/g) was added remove the Fmoc protecting group (deprotection), followed by nitrogen bubbling for 30 min. The resin was then washed six times with DMF, with each wash consisting of 1 min of bubbling and 1 min of solvent removal. Subsequently, 3 equivalents of Fmoc-Dab (Boc)-OH were dissolved in DMF with OXYMA, followed by the addition of 3 equivalents of DIC. After 5 min of activation, nitrogen bubbling was applied for 1-2 h to complete the condensation reaction, yielding Fmoc-Dab-Leu-CTC resin. The residual solvent was removed by washing 4 times, and the solubilization, Fmoc deprotection and detection were repeated. Finally, another round of condensation was performed using 3 equivalents of Fmoc-Dab(Boc)-OH, OXYMA, and DIC activation solutions, followed by nitrogen bubbling for 1–2 h, resulting in the formation of Fmoc-Dab-Dab-Leu-CTC resin.

The amino acids (Fmoc-Ile-OH, Fmoc-DLeu-OH, Fmoc-Dab(Boc)-OH, Fmoc-Dab(ivdde)-OH, Fmoc-D-Dab(Boc)-OH, Fmoc-Thr(*t*Bu)-OH, and Fmoc-Dab(Boc)-OH) were sequentially condensed from right to left to synthesize the Fmoc-Dab-Thr-{D-Dab}-Dab-Dab-{D-Leu}-Ile-Dab-Dab-Leu-CTC resin. After repeated solubilization and deprotection, 3 equivalents of biphenyl and OXYMA were dissolved in DMF, followed by the addition of 3 equivalents of DIC for 5 min of activation. The reaction mixture was subjected to nitrogen bubbling for 1-2 h to complete the condensation. Then, the resin was washed 4 times with DMF, with each wash consisting of 1 min of bubbling and 1 min of solvent removal. For amino acid No. 5, Fmoc-DAB(Ivdde)-OH, side-chain deprotection was performed by treating the resin with 5% hydrazine hydrate in DMF (12 mL/g) for 3 times for 10 min each. Cyclization was achieved by dissolving 2 equivalents of HATU and 4 equivalents of DIEA in DMF, followed by nitrogen bubbling for 1–2 h. The final antimicrobial peptide was then washed and drained, undergoing 5 washes with DMF, 5 washes with methanol, and vacuum drying for 1 h. The dried peptide was then treated with 10 mL/g of lysis solution for 3h at room temperature, after which the reaction mixture was filtered. To precipitate the peptide, 10 times the volume of ice-cold ether was added, followed by centrifugation and 3 washes with ice-cold ether, yielding the crude white peptide product.

The purification of NP001 was performed using 0.065% TFA in warer as mobile phase A and 0.05% TFA acetonitrile solution as mobile phase B. Separation was conducted on a C18 column at a flow rate of 15 mL/min at room temperature. The peaks at 220 nm were detected, and fractions were collected for mass spectrometry analysis, including samples taken before the highest peak, at the highest peak, and after the peak. NP001 was then dissolved in water to a final concentration of 1 mg/mL and analyzed using mass spectrometry in positive ion mode on an SHIMADZU LCMS-2020 instrument. The mass spectrometry results provided the mass-to-charge ratio (m/z), where m represents molecular weight and z represents charge. The molecular weight of the purified product was determined by multiplying the mass-to-charge ratio by the charge state.

#### Generation of *MCR-1* strains

The *MCR-1* gene fragment (NCBI accession: CP100017.1, 23338–24963 bp region) was synthesized and cloned into the pET28a (+) plasmid by GenScript Biotech Ltd. The accuracy of the inserted sequence was confirmed by Sanger sequencing. The recombinant plasmid was then transformed into *E. coli* BL21, and *MCR-1* protein expression was induced by adding IPTG to a final concentration of 20 μm/L.

The *MCR-1* fragment was amplified from the pET28a-*MCR-1* plasmid, and the pBBR1MCS2 vector backbone was amplified using primers containing the corresponding homologous arms for recombination (Table S4). The purified *MCR-1* insert and linearized pBBR1MCS2 backbone were then assembled using a homologous recombination cloning kit according to the manufacturer’s instructions (C115, Vazyme Biotech Co., Ltd.). The recombinant products were transformed into *E. coli* DH5α competent cells, and positive colonies were selected for plasmid extraction. Successful construction of the pBBR1MCS2-*MCR-1* plasmid was confirmed by Sanger sequencing.

Electrocompetent cells of *E. coli* BL21(DE3), *P. aeruginosa* 29260, and *A. baumannii* 19606 were prepared as previously described.^21^^,^^22^ Briefly, pick single bacterial colonies and incubate them at 37°C and 220 rpm for 16 h. Next, the cultures were inoculated at a 1:200 dilution and re-activated. When the OD_600_ reached 0.6, the cells were centrifuged at 12,000 g at 4°C for 10 min and washed three times with pre-chilled glycerol for later use. Next, 1 μL of the pET28a-mcr-1 plasmid was mixed on ice with *E. coli* BL21(DE3), and 1 μL of the pBBR1MCS2-mcr-1 plasmid was mixed on ice with *P. aeruginos*a 29260 and *A. baumannii* 19606, and the mixtures were allowed to stand for 10 min. Transfer the mixture to a pre-chilled electroporation cup, insert it into the electroporation device, and perform electroporation using the following settings: 25 AF; 200 V; 2.5 kV. Quickly add 1,000 μL of LB liquid medium to the electroporation cup. After resuspending the cells, transfer the mixture to a 1.5-mL centrifuge tube and incubate at 37°C for 1 h. Finally, spread the transformation products of the pET28a-mcr-1 plasmid with *E. coli* BL21 (DE3) onto K^+^-selective plates, while the pBBR1MCS2-mcr-1 plasmid and the transformation products of *P. aeruginos*a 29260 and *A. baumannii* 19606 were plated on plates containing polymyxin (20 μg/mL) as a resistance marker and incubated at 37°C for 16 h. Single colonies were picked separately, verified by MIC testing, and stored for future use.

#### Antibacterial assays

Single colonies of the bacteria to be tested were inoculated into 5 mL of medium and incubated overnight until saturation. The bacterial concentration was adjusted to 5∗10^5^ CFU/mL according to ISO standards. The antimicrobial peptides were dissolved to the final concentration of 0.5–512 μg/mL by 10 consecutive dilutions according to the 2-fold serial dilution method. Eleven gradients of antimicrobial peptide diluted to obtain 50 μL, followed by 50 μL of diluted bacteria, were added to each of the 96-well plates and mixed well. After incubating for 16 h at 37°C celsius, the OD600 of the bacteria was measured to determine which concentration at which the bacteria did not grow at all was the minimum inhibitory concentration.

Bacterial solution (0.5 McFarland) was applied to the antibiotic-free solid LB plate and left for 5 min for complete absorption of the bacterial solution. The antibiotic-free paper tablet was picked up with sterile forceps and lightly pressed onto the agar surface. Pipette 5 μg of NP001 or Colistin onto the plate and leave for 5 min for complete absorption. Invert the plate and incubate at 37°C for 16–18 h, and counting the results.

#### Mouse infection models

All mouse experiments were conducted in accordance with ethical guidelines and approved by the Ethics Committee of Army Medical University (Ethics ID: AMUWEC20223336). To establish a neutropenic mouse model, six-week-old BALB/c female mice (18–22 g) were used in this experiment. Mice were randomly housed in individually ventilated cages and maintained according to the standards of the American Association for Laboratory Animal Science (AALAS). The housing environment was controlled with a 12-h light/dark cycle, a temperature of 21°C, and a humidity of 30%. To induce neutropenia, mice were injected intraperitoneally with cyclophosphamide at doses of 150 mg/kg on day 4 and 100 mg/kg on day 1 prior to bacterial infection.

A monoclonal clone of polymyxin-resistant *E. coli* BW20676 (*MCR-1*) was selected and cultured in LB medium at 37°C overnight. The cultures were centrifuged, the supernatant was removed, and the bacterial pellet was gently washed twice with sterile saline. The OD_600_ of the bacterial suspension was measured and adjusted through dilution. 50 μL of the bacterial suspension was injected into left thighs of the mice via subcutaneous injection, providing a bacterial load of approximately 1.0∗10^6^ cf.u per thigh. At 2-, 8-, 14-, and 20-h post-infection, mice received a 50 μL subcutaneous injection of either vector control (0.9% saline), Colistin (30 mg/kg), or NP001 (30 mg/kg). At 24 h post-infection, mice were euthanized via CO_2_ asphyxiation, and thigh muscles were aseptically excised, weighed, homogenized, and plated on LB agar for CFU enumeration to assess bacterial burden. Statistical analysis of graphical data was perform using GraphPad Prism (Prism v.9).

To induce neutropenia, mice were intraperitoneally injected with cyclophosphamide at doses of 150 mg/kg on day 4 and 100 mg/kg on day 1 prior to bacterial infection, following the same protocol used in the thigh skin infection model. For the lethal dose infection model, mice received an intraperitoneally injection of approximately 1.0 × 10^9^ cf.u of bacteria. For the semi-lethal dose model, an intraperitoneal injection of approximately 2.0 × 10^8^ cf.u was administered. At 2-, 8-, 14-, and 20- hours after post-infection, mice were treated with a 100 μL intraperitoneal injection of either vector control (0.9% saline), Colistin (10 mg/kg), or NP001 (10 mg/kg). At 24 h post-infection, mice were euthanized via CO2 asphyxiation, and liver, spleen, and kidney tissues were aseptically excised, weighed, homogenized, and plated on LB agar for CFU enumeration to assess bacterial burden. All graphical data were statistically analyzed using GraphPad Prism software (Prism v.9).

#### Nephrotoxicity assessment

Six-week-old Balb/C female mice (18–22 g) were used for this experiment. The mice were housed under controlled conditions, with a 12-h light/dark cycle, a temperature of 21°C, and a humidity of 30%. After 3 days of acclimatization, mice were randomly divided into three groups (*n* = 5 per group): the carrier (0.9% saline) group, the Colistin group, and the NP001 group.

For subcutaneous injection experiment, mice received a 100 μL injection of 0.9% saline (carrier group), 30 mg/kg of Colistin, or 30 mg/kg of NP001once daily for 7 consecutive days. The mice’s body weight was measured and recorded daily. Blood samples were collected 12 h after the final injection, and serum NGAL concentrations were measured using a mouse NGAL ELISA kit to assess nephrotoxicity. Fresh mouse kidney tissue was fixed in a fixative solution for ≥24 h. After fixation, the tissue was removed, the target area was trimmed in a fume hood, and the specimen was placed in a correspondingly numbered embedding frame. The tissue was rinsed under running tap water for 20 min, then placed in a dehydrator for sequential gradient ethanol dehydration (75% ethanol for 2 h, 85% ethanol for 2 h, 95% ethanol for 1 h and 2 h, anhydrous ethanol for 1 h and 1 h). Subsequently, the tissue was cleared in xylene (1 h × 2 times) and immersed in molten paraffin (1 h and 2 h). After paraffin embedding was completed, sections were cut (3–5 μm). The sections were sequentially dewaxed using an eco-friendly dewaxing agent (10 min × 3 times), followed by stepwise rehydration (anhydrous ethanol, 95%, 85%, and 75% ethanol for 5 min each), and rinsed under running water for 1 min. Subsequently, the sections were stained with hematoxylin for 4 min, rinsed with tap water for 2 min, and then decolorized for a few seconds with 0.8% hydrochloric acid alcohol, followed by rebluing under running water or with a lithium carbonate solution, and washed with water for 2 min; stain with eosin for 20 s, followed by brief fixation in 95% ethanol (approximately 5 s), then dehydrate with anhydrous ethanol (2 min × 2 times), and finally clear with a clearing agent and mount with neutral resin. Observe under a microscope and take photographs.

For the intraperitoneal injection experiment, mice were administered a 100 μL injection of either 0.9% saline (carrier group), 10 mg/kg of Colistin, or 10 mg/kg of NP001 once daily for 7 days. Blood samples were collected 12 h after the final injection, and serum NGAL concentrations were measured using a mouse NGAL ELISA kit to assess nephrotoxicity.

#### Pharmacokinetic assays

Six-week-old Balb/C female mice (18–22 g) were used for this experiment. The mice were housed under controlled conditions, including a 12-h light/dark cycle, a temperature of 21°C, and a humidity of 30%. Following 3 days of acclimatization, pharmacokinetic experiments were conducted. For the subcutaneous administration, mice received a 30 mg/kg subcutaneous injection of NP001, and 100 μL of blood was collected via lateral tail vein puncture at 0.5, 1, 3, and 5 h post-administration. For the intraperitoneal administration, mice received a 10 mg/kg intraperitoneal injection of NP001, and 100 μL of blood was collected via lateral tail vein puncture at the same time points (0.5, 1, 3, and 5 h). Blood samples was centrifuged to isolate plasma, followed by protein precipitation with acetonitrile containing 0.1% TFA. The mixture was then centrifuged at 12,000 rpm for 15 min, and the supernatant was collected. The extraction was repeated once using the same volume of 80% acetonitrile containing 0.1% TFA. The combined supernatants were subsequently used for analysis.

Quantification of NP001 in plasma was performed using a Sciex Applied Biosystems Qtrap 6500+ Triple Quadrupole Mass Spectrometer, coupled to a Shimadzu Nexera *X*2 Ultra-High-Performance Liquid Chromatography (UHPLC) System. Chromatographic separation was carried out on a Phenomenex Luna Omega Polar C18 column (2.1 × 100 mm; particle size, 3 μm) using a reversed-phase gradient. Milli-Q deionized water with 0.1% formic acid served as mobile phase A, while acetonitrile with 0.1% formic acid served as mobile phase B. Multiple reaction monitoring (MRM) was employed in electrospray positive ionization mode to quantify analytes by monitoring parent-to-daughter ion transitions. Data were processed and analyzed using Analyst software (version 1.6.2; Applied Biosystems Sciex).

#### Molecular modeling

The PDB file containing Kdo2-lipidA (PDB: 3FXI) and Colistin (PDB: 8DEV) were obtained from the RCSB Protein DataBank (https://www.rcsb.org/). The molecular structures of Kdo2-lipidA and Colistin were subsequently extracted from their respective files for further analysis.

Based on the predicted amino acid composition and conformation of NP001, its 2D structure was drawn using ChemDraw. The preliminary 3D structure was then optimized in Chem3D using the MMFF94 semi-empirical force field. Further optimization of the 3D structure was performed using Gaussian 16, employing density flooding theory (DFT) calculations with the B3LYP/6-31g (d) basis set.

The structure-optimized phosphoethanolamine (PEA) (following the same structure optimization process as NP001) was conjugated to the phosphoryl group at position 1 of Kdo2-LipidA, resulting in the construction of the 3D structure of 1-PEA-Kdo 2-Lipid A. The molecular interactions between Colistin and Kdo2-lipidA, NP001 and Kdo2-Lipid A, and NP001 and 1-PEA-Kdo 2-Lipid A were visualized and analyzed using PyMOL.

#### Domain structure comparison

The 3D structures of the 350 C domains and the 350 T domains, which were used to construct the phylogenetic tree, were individually predicted using AlphaFold3 (https://alphafoldserver.com/). Among the generated models, Model 0 was selected as the input file for structure comparison. To assess structural similarity, TM scores of paired C and T domain structures were calculated using USalign (Version 20241108, C Zhang, M Shine, AM Pyle, Y Zhang. (2022) Nat Methods; C Zhang, AM Pyle (2022) iScience.). The average TM scores of the paired structures were computed and subsequently converted into structural distances. These paired distances were then used to generate a distance matrix, which was visualized as a heatmap.

#### Lipid A extraction

*E. coli* BW20676 (*MCR*-1) and *E. coli* BL21 strains were cultured in LB broth at 37°C until OD_600_ reached approximately 1.2. Bacterial cells were collected by centrifugation and treated with a chloroform/methanol/water mixture (1:2:0.8, v/v/v) for 1 h at room temperature, followed by centrifugation at 2000 rpm for 20 min. The pellet was resuspended in 12.5 mM sodium acetate buffer (pH 4.5) and incubated at 100°C for 30 min to hydrolyze the core oligosaccharide. After cooling, a chloroform/methanol/water mixture (1.8:1:2, v/v/v) was added and mixed thoroughly. The organic phase (lower layer) was collected, concentrated by rotary evaporation, and the residue was dissolved in a chloroform/methanol mixture (4:1, v/v) to recover Lipid A. The final product was vacuum-dried and stored at −20°C for subsequent use.

#### ITC measurements

The binding interactions between *MCR*-1-modified Lipid A and NP001 or Colistin E were measured using a MicroCal Auto-iTC200 calorimeter. Lipid A extracted from BW20676 (*MCR*-1) and *E. Coli* BL21 was dissolved in DMSO and diluted to 10 μM using PBS. NP001 and colistin E were dissolved in the same buffer at a concentration of 400 μm. During titration, 0.4 μL of NP001 or colistin E was injected into the sample cell, with a total of 20 injections. Each injection lasted 0.8 s, with an interval of 150 s between consecutive injections. The titration data were analyzed using Origin 7.0 software.

#### Transmission electron microscopy

*E. coli* BW20676 (*MCR*-1) and *E. coli* BL21 strains were cultured in LB broth at 37°C. Bacterial suspensions were adjusted to a concentration of 1 × 10^8^ CFU/mL and incubated with 5 μg/mL of NP001 or colistin E for 8 h. Cells were collected by centrifugation, washed twice with PBS, and resuspended to an appropriate concentration. The bacterial samples were fixed with glutaraldehyde fixative solution overnight at 4°C. After fixation, the samples were rinsed with 0.1 M phosphate buffer (PB) and post-fixed with 2% osmium tetroxide containing 1.5% potassium ferrocyanide in the dark for 1 h. Subsequently, the samples were dehydrated through a graded ethanol and propylene oxide series, followed by gradual infiltration and embedding in Epon812 resin. Ultrathin sections were prepared, stained with uranium and lead, and observed under a Talos L120C transmission electron microscope.

### Quantification and Statistical analysis

#### Statistical analysis

Statistical analysis of graphical data was performed using GraphPad Prism (Prism v.9). All graphical data were statistically analyzed using GraphPad Prism software (Prism v.9).

CFU count in mouse thigh tissue following NP001 treatment (*n* = 18). ∗∗*p* < 0.01, ∗∗∗∗*p* < 0.0001, unpaired *t* test.

Plasma NGAL levels after subcutaneous injection of NP001, Colistin, and Saline (*n* = 5). Ns, *p* > 0.05, ∗∗∗*p* < 0.001, ∗∗∗∗*p* < 0.0001, unpaired *t* test.

CFU count in the liver, spleen, and kidney of mice following antibiotic treatment (*n* = 10). ∗∗*p* < 0.01, ∗∗∗*p* < 0.001, ∗∗∗∗*p* < 0.0001, unpaired *t* test.

Plasma NGAL levels after intraperitoneal injection of NP001, Colistin, and Saline (*n* = 5). ∗*p* < 0.05, ∗∗*p* < 0.01, unpaired *t* test.

## Supplemental information

Document S1. Figures S1–S7 and Tables S2–S4

Table S1. Integrated antimicrobial peptide sequences corresponding to the BGCs, including 115 complete and 134 incomplete peptides, related to Figure 2

Table S5. Strain information and NCBI accession numbers for *Bacillus* and *Paenibacillus* strains used in antimicrobial peptide prediction, related to Figures 1 and 2

Document S2. Article plus supplemental information
