# Supplementary material for: Discovery of antimicrobial peptides from incomplete biosynthetic gene clusters to combat multidrug-resistant bacteria
Source: Cell Rep Med. 2026 Aug 12;7(9):102982. doi: 10.1016/j.xcrm.2026.102982 (PMC13589470; doi:10.1016/j.xcrm.2026.102982)
Supplement: Supplementary file 1 [file mmc1.pdf]

Cell Reports Medicine, Volume *7*

Supplemental information

Discovery of antimicrobial peptides from incomplete biosynthetic gene clusters to combat multidrug-resistant bacteria

Yao Tang (唐瑶), Xin Cheng (程新), Jing Zhou (周晶), Yuqi Shi (石宇旗), Jiaying Zhou (周佳颖), Zaiyang Chen (陈再洋), Yan Zhang (张艳), Liucun Zhu (朱柳村), Jian-Qun Chen (陈建群), Jiang Gu (顾江), Qiang Wang (王强), and Qihan Chen (陈奇涵)

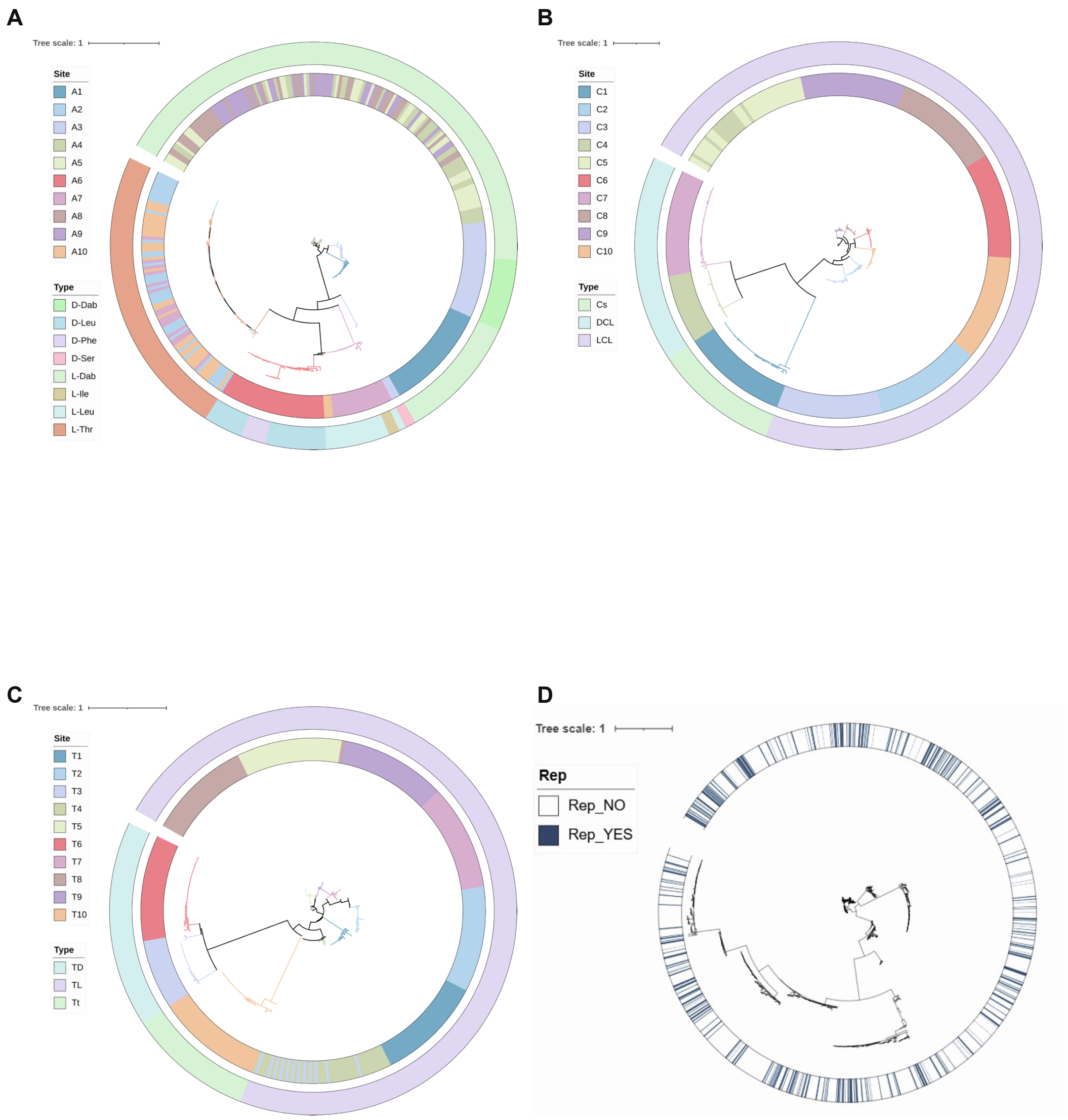

Figure S1 Integrated phylogenetic analysis of A, C, and T domains and representative BGC-containing species. Related to Figure 1. (A) Phylogenetic Tree of A Domains from Polymyxin-like BGCs. The outer ring represents the types of amino acids, while the inner ring indicates their positions within the modules. (B) Phylogenetic Tree of C Domains from Polymyxin-like BGCs. The outer ring represents the types of domain, while the inner ring indicates the positions of amino acids within the modules. Cs (C_Starter) catalyzes the formation of a peptide bond between the carboxyl group of a

fatty acid and the amino group of the first L-amino acid in the peptide. DCL catalyzes the formation of a peptide bond between the carboxyl group of a D-amino acid and the amino group of an L-amino acid. LCL catalyzes the formation of a peptide bond between the carboxyl group of an L-amino acid and the amino group of another L-amino acid. (C) Phylogenetic Tree of T Domains from Polymyxin-like BGCs. The outer ring represents the types of domain, while the inner ring indicates the positions of amino acids within the modules. TD transfers D-amino acid-peptide intermediates to the next module. TL transfers L-amino acid-peptide intermediates to the next module. Tt (*T_Terminal*) transfers the final peptide product to the TE domain. (D) The phylogenetic tree of all identified C-A-T unit in this study. The tree was constructed using the three domains (C, A, T) as a single unit. Sequences color-coded in the ring are derived from 35 BGCs, while uncolored sequences are non-representative.

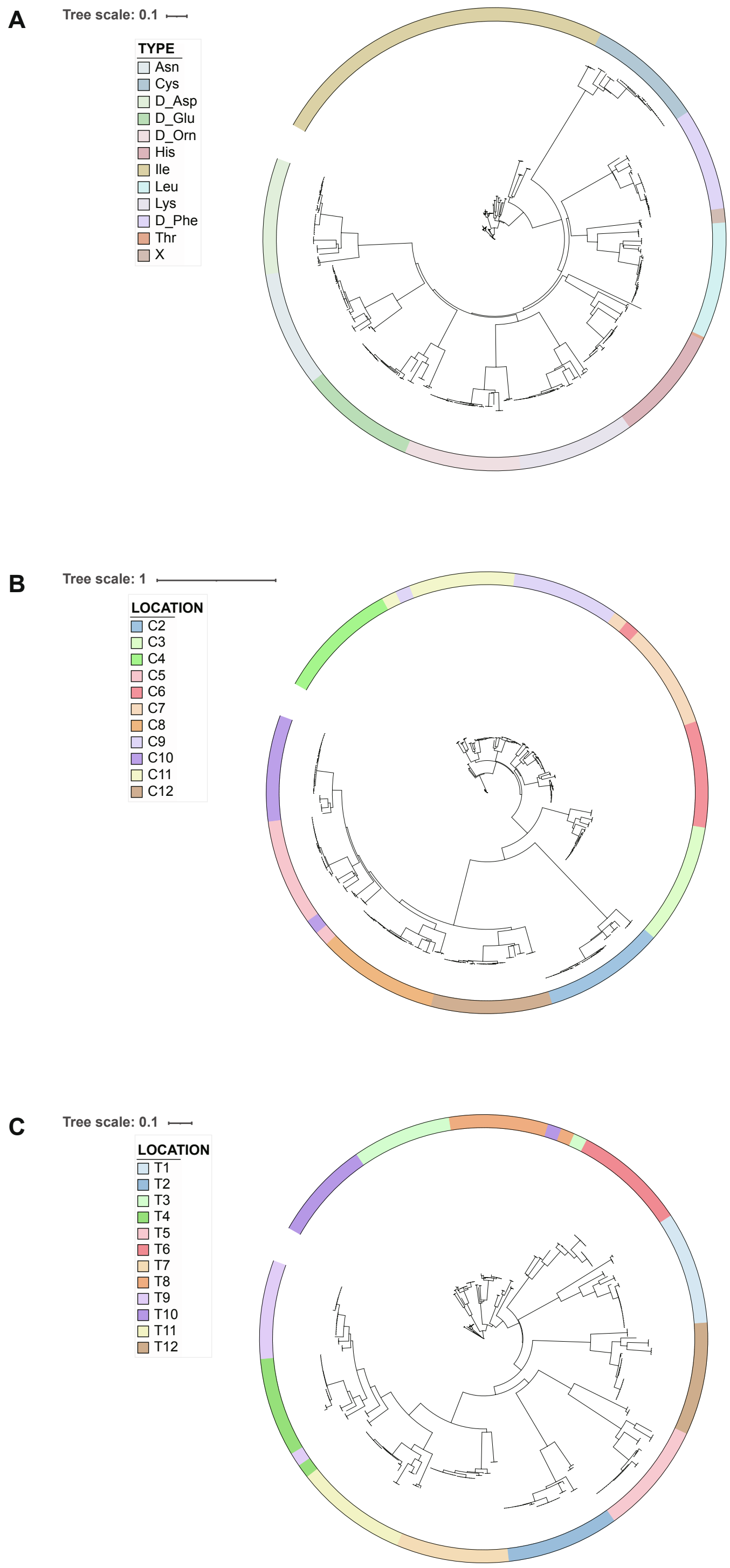

Figure S2 Phylogenetic Tree of A, C, T Domains from bacitracin BGCs. Related to Figure 1. A domain (A); C domain (B); T domain (C).

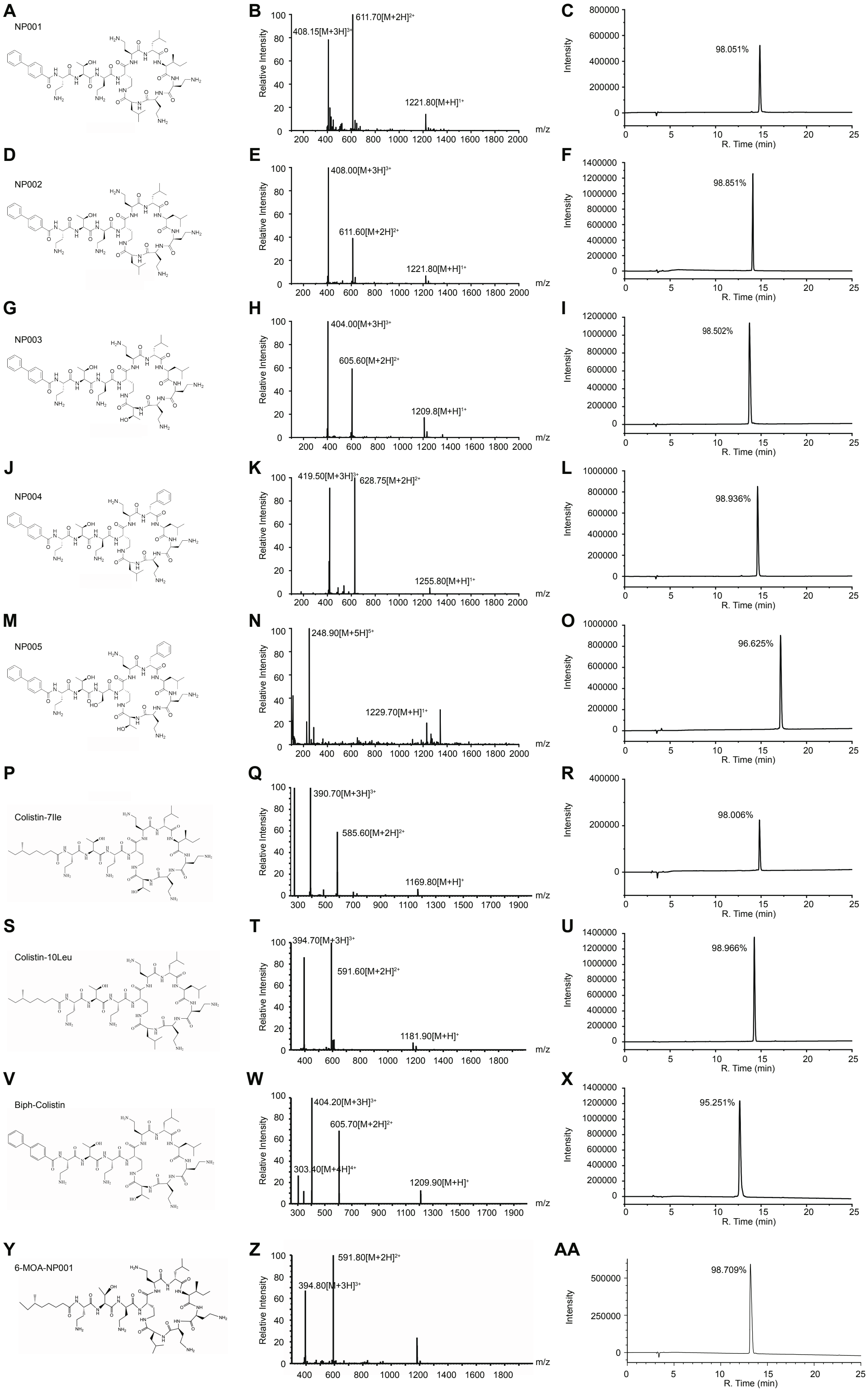

A
NP001
B
408.15[M+3H]3+
611.70[M+2H]2+
1221.80[M+H]1+
C
98.051%
D
NP002
E
408.00[M+3H]3+
611.60[M+2H]2+
1221.80[M+H]1+
F
98.851%
G
NP003
H
404.00[M+3H]3+
605.60[M+2H]2+
1209.8[M+H]1+
I
98.502%
J
NP004
K
419.50[M+3H]3+
628.75[M+2H]2+
1255.80[M+H]1+
L
98.936%
M
NP005
N
248.90[M+5H]5+
1229.70[M+H]1+
O
96.625%
P
Colistin-7Ile
Q
390.70[M+3H]3+
585.60[M+2H]2+
1169.80[M+H]+
R
98.006%
S
Colistin-10Leu
T
394.70[M+3H]3+
591.60[M+2H]2+
1181.90[M+H]+
U
98.966%
V
Biph-Colistin
W
404.20[M+3H]3+
605.70[M+2H]2+
303.40[M+4H]4+
1209.90[M+H]+
X
95.251%
Y
6-MOA-NP001
Z
591.80[M+2H]2+
394.80[M+3H]3+
AA
98.709%
Relative Intensity
m/z
Intensity
R. Time (min)

Figure S3 Structure, Mass, and Purity Characterization of Antimicrobial Peptides. Related to Figure 2 and 6. The antimicrobial peptides are arranged from top to bottom and displaying their structure (A, D, G, J, M, P, S, V, Y), molecular weight (B, E, H, K, N, Q, T, W, Z), and product purity by HPLC analysis (C, F, I, L, O, R, U, X, AA). Details on the identification and analysis procedures can be found in the Methods section.

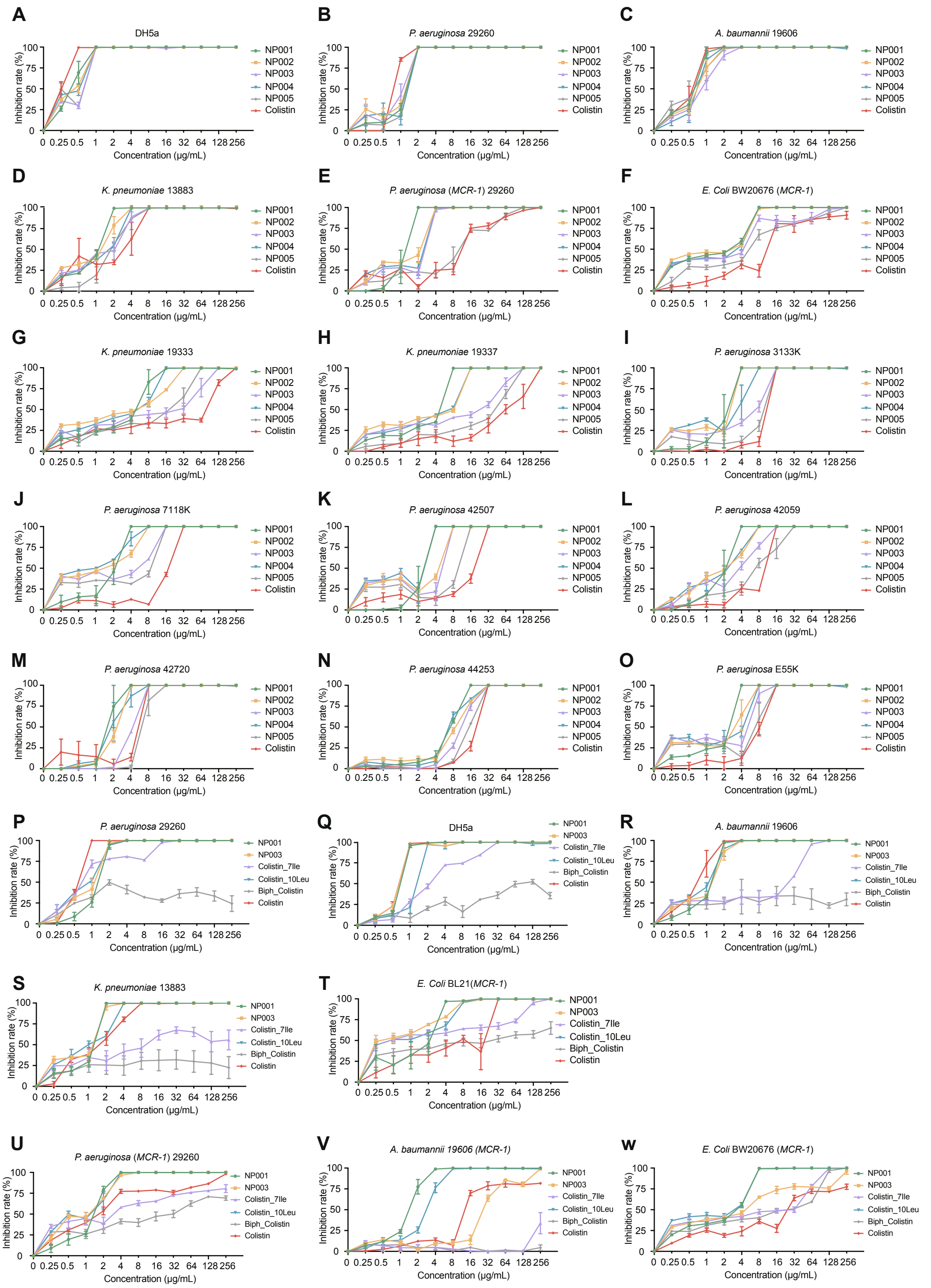

Figure S4 Inhibitory Effects of Antimicrobial Peptides. Related to Figure 3 and 6. Bacterial treatment and cultivation were conducted following the standard MIC method, and bacterial concentration was measured using OD600. Detailed calculations of inhibitory efficiency can be found in the Methods section. The x-axis represents the final antibiotic concentration, while the y-axis indicates the bacterial inhibition efficiency. All MIC assays were performed with three biological replicates. Erro bar represented SEM.

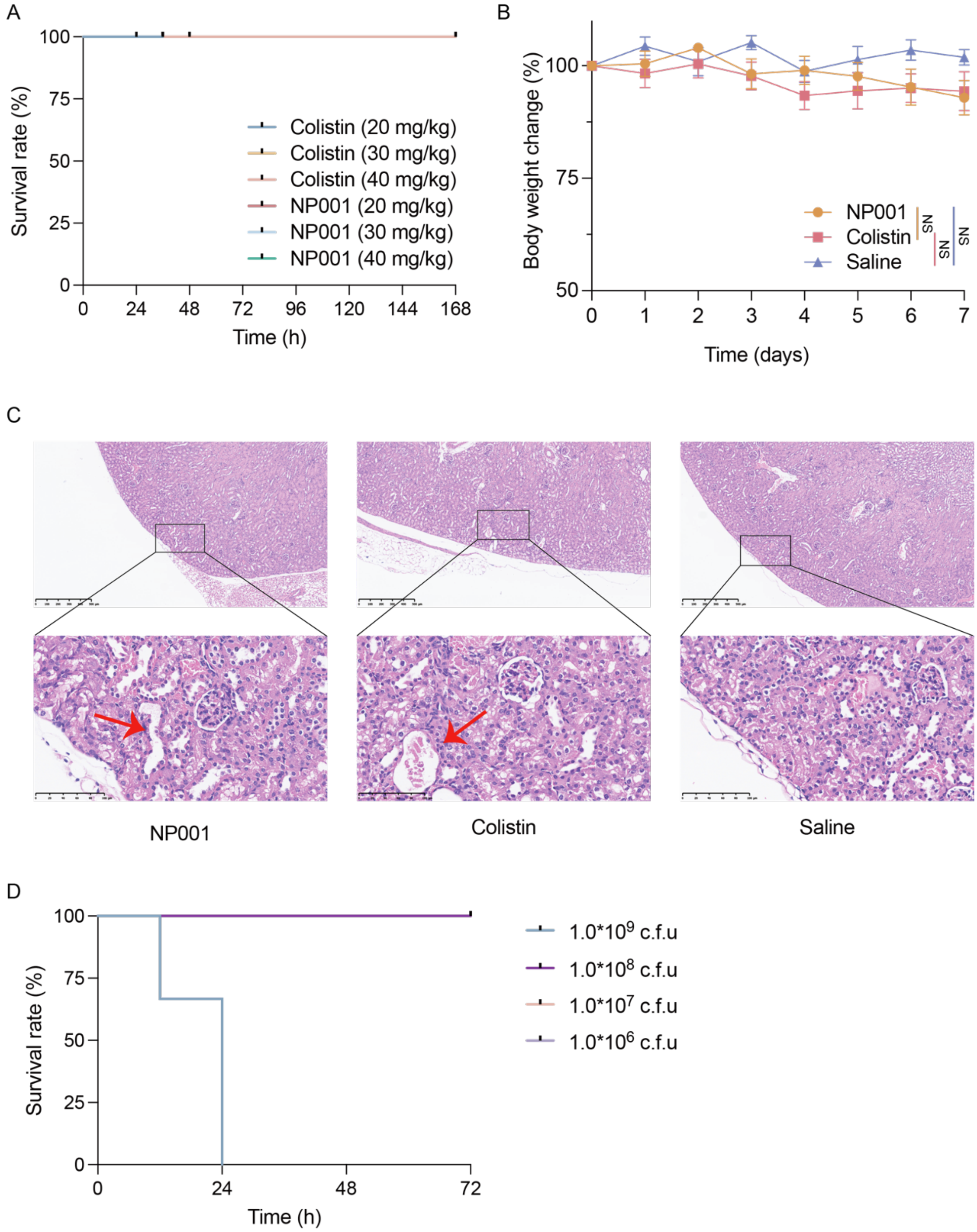

Figure S5 Safety evaluation and dosing optimization of NP001 in mouse infection models. Related to Figure 4 and 5. (A) Determination of safe dosage for subcutaneous injection (*n* = 5, biological replicates). (B) Changes in mouse body weight after drug administration (*n* = 5, biological replicates). (C) H&E staining of mouse kidneys after drug administration. The arrow indicates the injured renal tubule. Scale bars, 500 μm in the upper images and 100 μm in the lower images. (D) Optimization of bacterial dosing in bacterial peritonitis (*n* = 5, biological replicates).

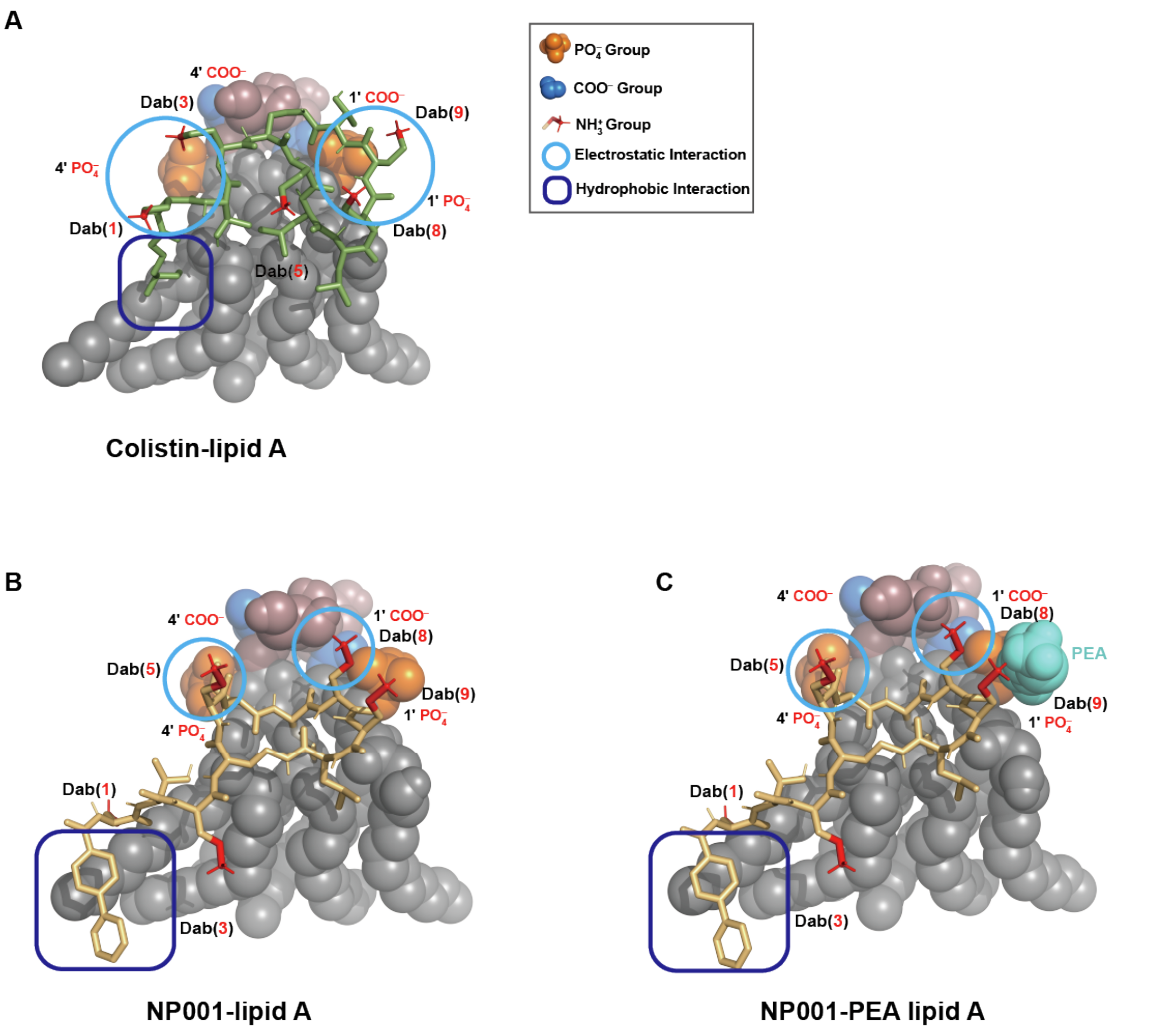

D

	6-MOA_NP001	Biph_NP001	Colistin
E. coli **BW20676 (MCR-1)**	16	8	128
K. pneumoniae **19333**	16	16	128
K. pneumoniae **19337**	8	8	128
P. aeruginosa **3133K**	2	8	32
P. aeruginosa **7118K**	4	2	32
P. aeruginosa **42059**	8	8	16
P. aeruginosa **42720**	8	4	8
P. aeruginosa **44253**	32	32	32
P. aeruginosa **E55K**	32	8	64

Figure S6 Analysis of antimicrobial peptide interactions with lipid A and the effect of N-terminal acyl substitution on NP001 activity. Related to Figure 6. (A) Schematic representation of polymyxin E interaction with Kdo$_2$-lipid A. Blue circles indicate electrostatic interactions between the positively charged amino acids of the antimicrobial peptide and the negatively charged phosphate or carboxyl groups on lipid A. Purple circles represent hydrophobic interactions between the fatty acyl chains of the peptide and lipid A. (B) Schematic representation of NP001 interaction with Kdo$_2$-lipid A. (C) Schematic representation of NP001 interaction with *mcr-1*-modified Kdo$_2$-lipid A-PEA. (D) MIC comparison of biphenyl-NP001 and 6-MOA-NP001 against the indicated bacterial strains.

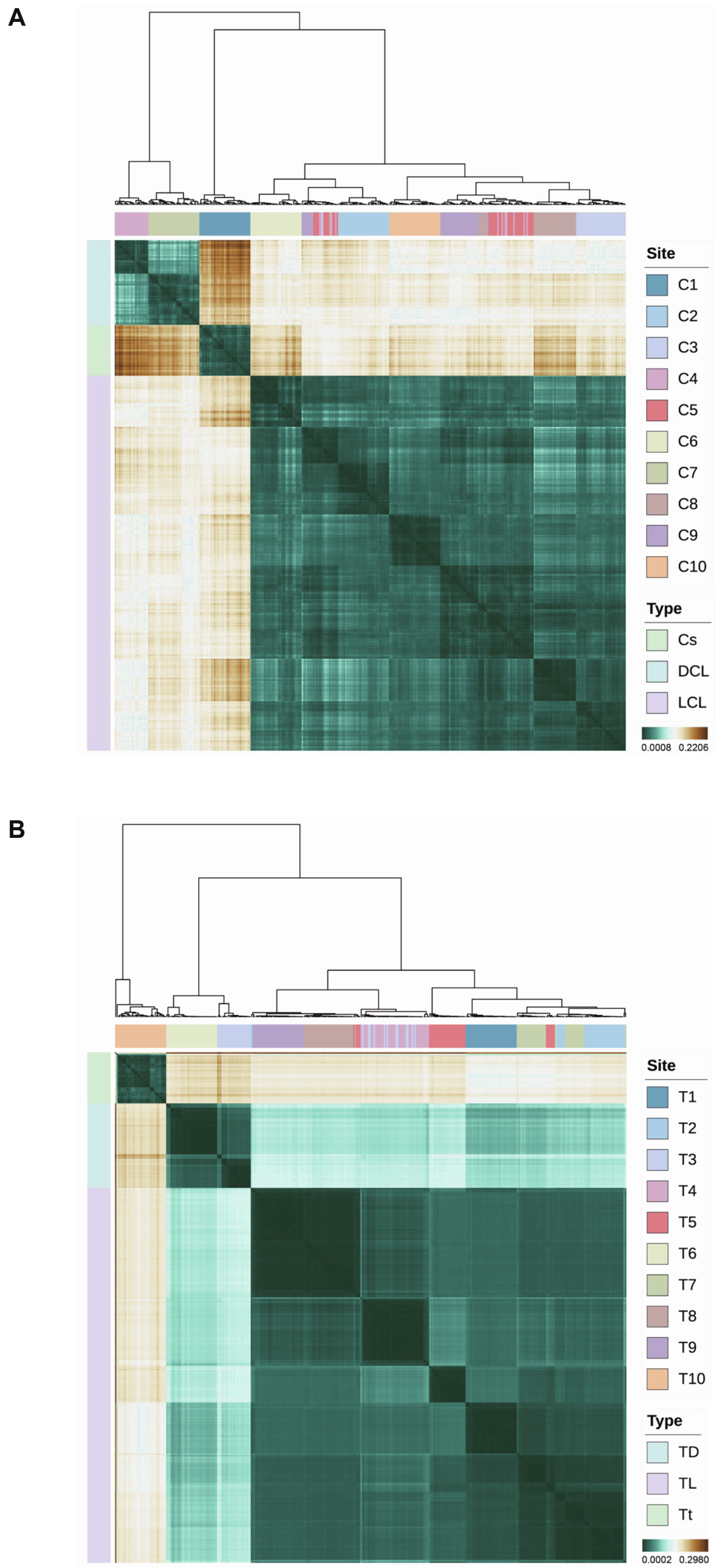

Figure S7 Three-dimensional structural clustering results of all representative C/T domains. Related to Figure 1. The three-dimensional structures of the C and T domains were predicted using AlphaFold3. C domain (A); T domain (B).

Table S2 Antimicrobial peptide types identified by the evolution-guided algorithm after removal of duplicates. Related to Figure 2. Only the four variable amino acid positions, A3, A6, A7, and A10, are showed here.

A3	A6	A7	A10	Index	Product
D-Dab	D-Leu	Thr	Thr	D-DabD-LeuThrThr	A
Dab	D-Phe	Leu	Thr	DabD-PheLeuThr	B
D-Dab	D-Phe	Leu	Thr	D-DabD-PheLeuThr	B-Dab3
D-Ser	D-Leu	Thr	Thr	D-SerD-LeuThrThr	D
Dab	D-Leu	Leu	Thr	DabD-LeuLeuThr	E
D-Dab	D-Phe	Thr	Thr	D-DabD-PheThrThr	P
D-Ser	D-Leu	Ile	Leu	D-SerD-LeuIleLeu	Macolacin
Dab	D-Phe	Ile	Thr	DabD-PheIleThr	B-Ile
Dab	D-Leu	Ile	Thr	DabD-LeuIleThr	E-Ile
Dab	D-Leu	Thr	Thr	DabD-LeuThrThr	M
D-Ser	D-Phe	Thr	Thr	D-SerD-PheThrThr	S
Dab	D-Phe	Leu	Leu	DabD-PheLeuLeu	T
D-Dab	D-Leu	Ile	Leu	D-DabD-LeuIleLeu	NP001
D-Dab	D-Leu	Leu	Leu	D-DabD-LeuLeuLeu	NP002
D-Dab	D-Leu	Leu	Thr	D-DabD-LeuLeuThr	NP003
D-Dab	D-Phe	Leu	Leu	D-DabD-PheLeuLeu	NP004
D-Ser	D-Phe	Leu	Thr	D-SerD-PheLeuThr	NP005

Table S3 Antimicrobial peptide products identified by antiSMASH in *Paenibacillus* species, related to Figure 1 and 2.

Genome	Antismash_Product
P_alvei_GCF_900519125_1	NP003
P_amylo_GCF_029542105_1	Macolacin
P_barci_GCF_013347305_1	NP001
P_dendr_GCF_021654795_1	A
P_dendr_GCF_029952845_1	A
P_dendr_GCF_945605565_1	A
P_lentus_GCF_003931855_1	NP003
P_peori_GCF_001272655_2	A
P_peori_GCF_014692735_1	A
P_peori_GCF_022531965_1	P
P_polym_ATCC_842_GCF_022811565_1	E
P_polym_E681_GCF_000146875_3	A
P_polym_E681_GCF_014706555_1	A
P_polym_GCF_000819665_1	B-Dab3
P_polym_GCF_001719045_1	B
P_polym_GCF_001874405_2	D
P_polym_GCF_001874425_3	A
P_polym_GCF_001922145_1	A
P_polym_GCF_002893885_1	E
P_polym_GCF_006274405_1	E
P_polym_GCF_014854715_1	B-Dab3
P_polym_GCF_015710815_1	E
P_polym_GCF_015710975_1	E
P_polym_GCF_020181695_1	E
P_polym_GCF_022492955_1	E
P_polym_GCF_022649565_1	E
P_polym_GCF_023374295_1	E
P_polym_GCF_023586785_2	E
P_polym_GCF_024584565_1	E
P_polym_GCF_025720855_1	E
P_polym_GCF_028471985_1	A
P_polym_GCF_028751565_1	B-Dab3
P_polym_GCF_029203955_1	E
P_polym_M1_GCF_000237325_1	P
P_polym_SC2_GCF_000164985_3	P
P_thiam_GCF_007066085_1	A
P_thiam_GCF_028226775_1	A
P_xylane_GCF_001908275_1	Macolacin
P_sp_37_GCF_008386395_1	NP003
P_sp_DR_GCF_019793795_1	NP003

P_sp_IHB__GCF_001447315_1	A
P_sp_lz_GCF_002872435_1	B-Dab3
P_sp_M__GCF_003856555_1	B-Dab3
P_sp_S02_GCF_019454065_1	D
P_sp_S25_GCF_019454085_1	D
P_alvei_DSM_29_GCF_000293805_1	A
P_alvei_GCF_011009465_1	A
P_alvei_GCF_026768265_1	E
P_alvei_GCF_026794095_1	A
P_alvei_GCF_026797385_1	A
P_alvei_GCF_026797595_1	A
P_alvei_GCF_026797655_1	A
P_amylo_GCF_001956035_1	Macolacin
P_amylo_GCF_004358835_1	NP003
P_amylo_GCF_023516485_1	NP003
P_amylo_GCF_900095775_1	NP003
P_aquis_GCF_012926515_1	NP003
P_aquis_GCF_900177815_1	NP003
P_aquis_GCF_900188525_1	NP003
P_areno_GCF_014841135_1	NP002
P_cucum_GCF_019748695_1	NP003
P_dendri_GCF_018403065_1	A
P_jamil_GCF_001477135_1	B-Dab3
P_jamil_GCF_003428495_1	E
P_melis_GCF_945318285_1	A
P_polym_GCF_000735775_1	E
P_polym_GCF_000943535_1	E
P_polym_GCF_000955925_1	E
P_polym_GCF_002894925_1	E
P_polym_GCF_002916985_1	A
P_polym_GCF_017589695_1	P
P_polym_GCF_029453445_1	E
P_polym_GCF_900406265_2	A
P_polym_GCF_900454525_1	E
P_polym_GCF_900454545_1	E
P_sambh_GCF_003233845_1	B-Dab3
P_silvae_GCF_014639235_1	NP003
P_wooso_GCF_009725165_1	NP003
P_xylane_GCF_003833245_1	NP003
P_xylane_GCF_007676685_1	NP003
P_xylane_GCF_007679855_1	NP003
P_xylane_GCF_007680605_1	NP003
P_xylane_GCF_017874615_1	Macolacin

P_xylane_GCF_019749275_1	NP003
P_sp_11_GCF_017833915_1	P
P_sp_CF0_GCF_900101225_1	NP003
P_sp_CFBP__GCF_014842675_1	NP003
P_sp_F4_GCF_002894905_1	B-Dab3
P_sp_FSL_R5_1_GCF_000517845_1	Macolacin
P_sp_JG_GCF_014203995_1	NP003
P_sp_Lo_GCF_024213565_1	B
P_sp_MYb63_GCF_002975315_1	NP003
P_sp_P2__GCF_029522175_1	E
P_sp_PA_GCF_000316035_1	Macolacin
P_sp_PvR1_GCF_017833155_1	D
P_sp_UA_GCF_008693195_1	NP003
P_sp_UK_GCF_021530955_1	B-Dab3
P_sp_UNC2_GCF_000686865_1	E

Table S4 Primers and gene sequences used in this study. Related to Figure 3.

MCS-plasmid-F	gcgttaatattttgttaaaattcgcg
MCS-plamid-R	agctgtttcctgtgtgaaattgtt
MCS-MCR-F	atttcacacaggaaacagctATGATGCAGCATACTTCTGTGTGG
MCS-MCR-R	ttttaacaaaatattaacgcTCAGCGGATGAATGCGGT
MCR-1 sequence	ATGATGCAGCATACTTCTGTGTGGTACCGACGCTCGGTCAGTCCGTTT<br>GTTCTTGTGGCGAGTGTTGCCGTTTTCTTGACCGCGACCGCCAATCT<br>TACCTTTTTGATAAAATCAGCCAAACCTATCCCATCGCGGACAATCT<br>CGGCTTTGTGCTGACGATCGCTGTCGTGCTCTTTGGCGCGATGCTACT<br>GATCACCACGCTGTTATCATCGTATCGCTATGTGCTAAAGCCTGTGTT<br>GATTTTGCTATTAATCATGGGCGCGGTGACCAGTTATTTTACTGACAC<br>TTATGGCACGGTCTATGATACGACCATGCTCCAAAATGCCCTACAGAC<br>CGACCAAGCCGAGACCAAGGATCTATTAAACGCAGCGTTTATCATGC<br>GTATCATTGGTTTGGGTGTGCTACCAAGTTTGCTTGTGGCTTTTGTTA<br>AGGTGGATTATCCGACTTGGGGCAAGGGTTTGATGCGCCGATTGGGC<br>TTGATCGTGGCAAGTCTTGCGCTGATTTTACTGCCTGTGGTGGCGTTC<br>AGCAGTCATTATGCCAGTTTCTTTCGCGTGCATAAGCCGCTGCGTAGC<br>TATGTCAATCCGATCATGCCAATCTACTCGGTGGGTAAGCTTGCCAGT<br>ATTGAGTATAAAAAAGCCAGTGCGCCAAAAGATACCATTTATCACGC<br>CAAAGACGCGGTACAAGCAACCAAGCCTGATATGCGTAAGCCACGC<br>CTAGTGGTGTTCGTCGTCGGTGAGACGGCACGCGCCGATCATGTCAG<br>CTTCAATGGCTATGAGCGCGATACTTTCCCACAGCTTGCCAAGATCG<br>ATGGCGTGACCAATTTTAGCAATGTCACATCGTGCGGCACATCGACG<br>GCGTATTCTGTGCCGTGTATGTTCAGCTATCTGGGCGCGGATGAGTAT<br>GATGTCGATACCGCCAAATACCAAGAAAATGTGCTGGATACGCTGGA<br>TCGCTTGGGCGTAAGTATCTTGTGGCGTGATAATAATTCGGACTCAAA<br>AGGCGTGATGGATAAGCTGCCAAAAGCGCAATTTGCCGATTATAAAT<br>CCGCGACCAACAACGCCATCTGCAACACCAATCCTTATAACGAATGC<br>CGCGATGTCGGTATGCTCGTTGGCTTAGATGACTTTGTCGCTGCCAAT<br>AACGGCAAAGATATGCTGATCATGCTGCACCAAATGGGCAATCACGG<br>GCCTGCGTATTTTAAGCGATATGATGAAAAGTTTGCCAAATTCACGCC<br>AGTGTGTGAAGGTAATGAGCTTGCCAAGTGCGAACATCAGTCCTTGA<br>TCAATGCTTATGACAATGCCTTGCTTGCCACCGATGATTTCATCGCTC<br>AAAGTATCCAGTGGCTGCAGACGCACAGCAATGCCTATGATGTCTCA<br>ATGCTGTATGTCAGCGATCATGGCGAAAGTCTGGGTGAGAACGGTGT<br>CTATCTACATGGTATGCCAAATGCCTTTGCACCAAAAGAACAGCGCA<br>GTGTGCCTGCATTTTTCTGGACGGATAAGCAAACTGGCATCACGCCA<br>ATGGCAACCGATACCGTCCTGACCCATGACGCGATCACGCCGACATT<br>ATTAAAGCTGTTTGATGTCACCGCGGACAAAGTCAAAGACCGCACC<br>GCATTCATCCGCTGA
